# CO‐Releasing Polyoxometalates Nanozyme with Gut Mucosal Immunity and Microbiota Homeostasis Remodeling Effects for Restoring Intestinal Barrier Integrity

**DOI:** 10.1002/advs.202500116

**Published:** 2025-03-13

**Authors:** Hongyang Lu, Qiang Zhou, Jiayu Li, Shengming Xu, Li Yu, Yinci Zhu, He Zhang, Chengge Shi, Tianci Zuo, Mengzhu Xu, Mingli Su, Yanmei Zhang, Rongdang Hu, Quazi T. H. Shubhra, Hui Deng, Xiaowen Hu, Xiaojun Cai

**Affiliations:** ^1^ School and Hospital of Stomatology Wenzhou Medical University Wenzhou 325027 China; ^2^ Ruian People's Hospital The Third Affiliated Hospital of Wenzhou Medical University Wenzhou 325016 China; ^3^ Institute of Chemistry University of Silesia in Katowice Szkolna 9 Katowice 40‐006 Poland

**Keywords:** anti‐inflammatory, antioxidant, CO therapy, gut microbiota, intestinal epithelial barrier, polyoxometalate nanozyme, single‐cell RNA sequencing

## Abstract

Disruption of the intestinal epithelial barrier, driven by imbalances in gut mucosal immunity and microbial homeostasis, is central to the onset and progression of inflammatory bowel disease (IBD). This study introduces a CO‐releasing polyoxometalates (POMs) nanozyme (PMC), synthesized by coordinating pentacarbonyl manganese bromide with molybdenum‐based POM nanoclusters. PMC demonstrates targeted accumulation at IBD‐affected sites, efficient scavenging of reactive oxygen species (ROS), and responsive CO release, resulting in multiple therapeutic effects. Extensive in vitro and in vivo studies have validated the exceptional capacity of PMC to repair intestinal barrier, attributed to their potent antioxidant and anti‐inflammatory properties, thereby achieving significant therapeutic efficacy in ulcerative colitis treatment. 16S rRNA sequencing indicated that PMC efficiently remodeled the gut microbiota composition. Single‐cell RNA sequencing indicates a reduction in pro‐inflammatory M1 macrophages, alongside suppressed ROS and inflammatory signaling pathways. Concurrently, an increase in reparative M2 macrophages and intestinal stem cells is observed, in addition to significant activation of the VEGF signaling pathway in macrophages and the NOTCH pathway in stem cells, underscoring the potential of PMC to restore immune balance and promote tissue repair. This study positions PMC as a promising, multifunctional therapeutic agent for IBD treatment owing to its robust intestinal barrier‐restoring capability.

## Graphical abstract

A CO‐releasing polyoxometalates nanozyme (PMC) is engineered by coordinating pentacarbonyl manganese bromide with molybdenum‐based polyoxometalates nanoclusters. PMC exhibits targeted accumulation at IBD sites, efficient ROS scavenging, and responsive CO release. These advantageous features facilitate the efficient restoration of intestinal barrier integrity by modulating gut mucosal immunity and remodeling microbiota homeostasis, achieving significant therapeutic efficacy in the treatment of ulcerative colitis.

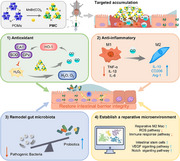

## 1 Introduction

Inflammatory Bowel Disease (IBD) is a chronic, non‐specific inflammatory disorder of the gastrointestinal tract^[^ ^1^ ^]^ that includes conditions such as Crohn's disease and ulcerative colitis (UC).^[^ ^2^ ^]^ These diseases are characterized by recurrent flare‐ups and pose significant challenges to both patients and healthcare systems worldwide.^[^ ^3^ ^]^ The increasing prevalence of IBD in recent years underscores the necessity for effective treatment options.^[^ ^4^ ^]^ Currently, the first‐line treatments for UC, such as 5‐aminosalicylic acid (5‐ASA), provide moderate relief by alleviating colonic inflammation. However, in severe cases, the efficacy of 5‐ASA is limited,^[^ ^5^ ^]^ and its long‐term use is associated with an elevated risk of nausea and headaches.^[^ ^6^ ^]^ Although corticosteroids (e.g., prednisone), Janus kinase inhibitors (e.g., tofacitinib), and biological therapies (e.g., infliximab) offer rapid anti‐inflammatory effects, their high costs, risks of systemic infections, and potential malignancy have restricted their widespread application.^[^ ^7^ ^]^ Consequently, the demand for safer and more effective IBD therapies is increasing.

The pathogenesis of IBD involves a complex interplay between genetic factors, immune dysregulation, and environmental triggers.^[^ ^8^ ^]^ A crucial factor in the initiation and exacerbation of IBD is the disruption of the intestinal epithelial barrier, which primarily consists of the intestinal epithelial cells, tight junctions, mucus layer, immune cells, and microbiota.^[^ ^9^ ^]^ This barrier is crucial for maintaining intestinal homeostasis by preventing the entry of harmful substances such as pathogens, toxins, and host antigens.^[^ ^10^ ^]^ When this barrier is compromised, the innate immune cells in the gut detect microbial invaders and release inflammatory mediators and chemokines^[^ ^11^ ^]^ that recruit additional immune cells, thereby triggering an inflammatory cascade.^[^ ^12^ ^]^ Activated macrophages produce reactive oxygen species (ROS), which combat pathogens.^[^ ^13^ ^]^ However, excessive ROS production can result in epithelial cell apoptosis via mechanisms such as lipid peroxidation, DNA damage, and protein dysfunction, thereby exacerbating damage to the intestinal barrier.^[^ ^14^ ^]^ Furthermore, ROS activate key pro‐inflammatory signaling pathways, such as nuclear factor‐kappa B (NF‐κB) and p38 MAPK,^[^ ^15^ ^]^ which amplify the expression of cytokines and chemokines.^[^ ^16^ ^]^ Elevated cytokine levels promote additional ROS production, thereby creating a self‐perpetuating cycle of “ROS‐cytokine‐ROS” that amplifies inflammation and accelerates intestinal damage.^[^ ^17^ ^]^ Additionally, pro‐inflammatory cytokines degrade tight junction proteins, resulting in increased intestinal permeability and irreversible structural damage to the intestinal barrier.^[^ ^18^ ^]^ This cycle contributes to the chronicity, progression, and frequent relapses of IBD. Given these challenges, the development of novel therapeutic approaches aimed at targeting ROS, modulating inflammation, and restoring intestinal barrier integrity has become a critical area of research.

Nanozymes have gained traction as promising enzyme‐mimicking materials for managing diseases associated with oxidative stress.^[^ ^19^ ^]^ Molybdenum (Mo)‐based POMs—Keggin‐type anion clusters composed of Mo and O atoms—have garnered significant attention owing to their potent ROS‐scavenging abilities against superoxide anions, hydrogen peroxide, and hydroxyl radicals.^[^ ^20^ ^]^ Notably, POMs show significant potential as targeted drug delivery vehicles for colonic therapies owing to their high surface area and distinctive heteropoly acid structure, which provides numerous binding sites for drug molecules and enhances their loading capacity.^[^ ^21^ ^]^ The distinct structure of POM, characterized by their highly negative charge density and the presence of multiple oxygen atoms, enables them to effectively coordinate with metal centers. This coordination capability allows POMs to interact with metal‐containing CO donors, facilitating the stable formation of complexes. Under the acidic conditions typical of inflamed tissues, POMs undergo self‐assembly into larger particles, which enhances their retention and prolongs their therapeutic effects.^[^ ^22^ ^]^ Despite their significant potential for IBD treatment, the efficacy of POMs is size‐dependent, with smaller particles demonstrating superior ROS‐scavenging capabilities.^[^ ^23^ ^]^ Furthermore, their ROS‐scavenging activity also relies on the conversion of Mo⁵⁺ to Mo⁶⁺, which reduces their efficacy as Mo⁵⁺ is depleted.^[^ ^24^ ^]^ Additionally, the single‐function nature of ROS clearance by POMs may likely be insufficient to address the complex chronic inflammation characteristic of IBD. Therefore, combining POMs with other therapeutic agents to enhance their antioxidant, anti‐inflammatory, and microbiota‐modulating properties is a promising strategy for effectively repairing the intestinal barrier.

To address these challenges, we propose a novel therapeutic strategy that combines POMs with CO gas therapy to augment the antioxidant, anti‐inflammatory, and microbiota‐modulating properties of POMs.^[^ ^25^ ^]^ This strategy is based on several key insights: 1) CO functions as a crucial endogenous signaling molecule, playing significant roles in both physiological and pathological processes.^[^ ^26^ ^]^ 2) CO exerts potent antioxidant effects by activating the heme oxygenase‐1 (HO‐1) signaling pathway, downregulating ROS‐related gene expression, and concurrently modulating inflammation via selective inhibition or activation of specific pro‐ and anti‐inflammatory pathways.^[^ ^27^ ^]^ 3) CO enhances the bactericidal activity of macrophages against pathogenic bacteria, thereby mitigating intestinal inflammation.^[^ ^28^ ^]^ 4) CO promotes epithelial cell restitution and stimulates fibroblast growth factor production from myofibroblasts, thereby maintaining mucosal integrity.^[^ ^29^ ^]^ 5) for effective IBD treatment, precise delivery of CO donors to inflamed sites is essential, with POMs serving as an optimal carrier owing to their unique structural properties.

**Scheme 1:**
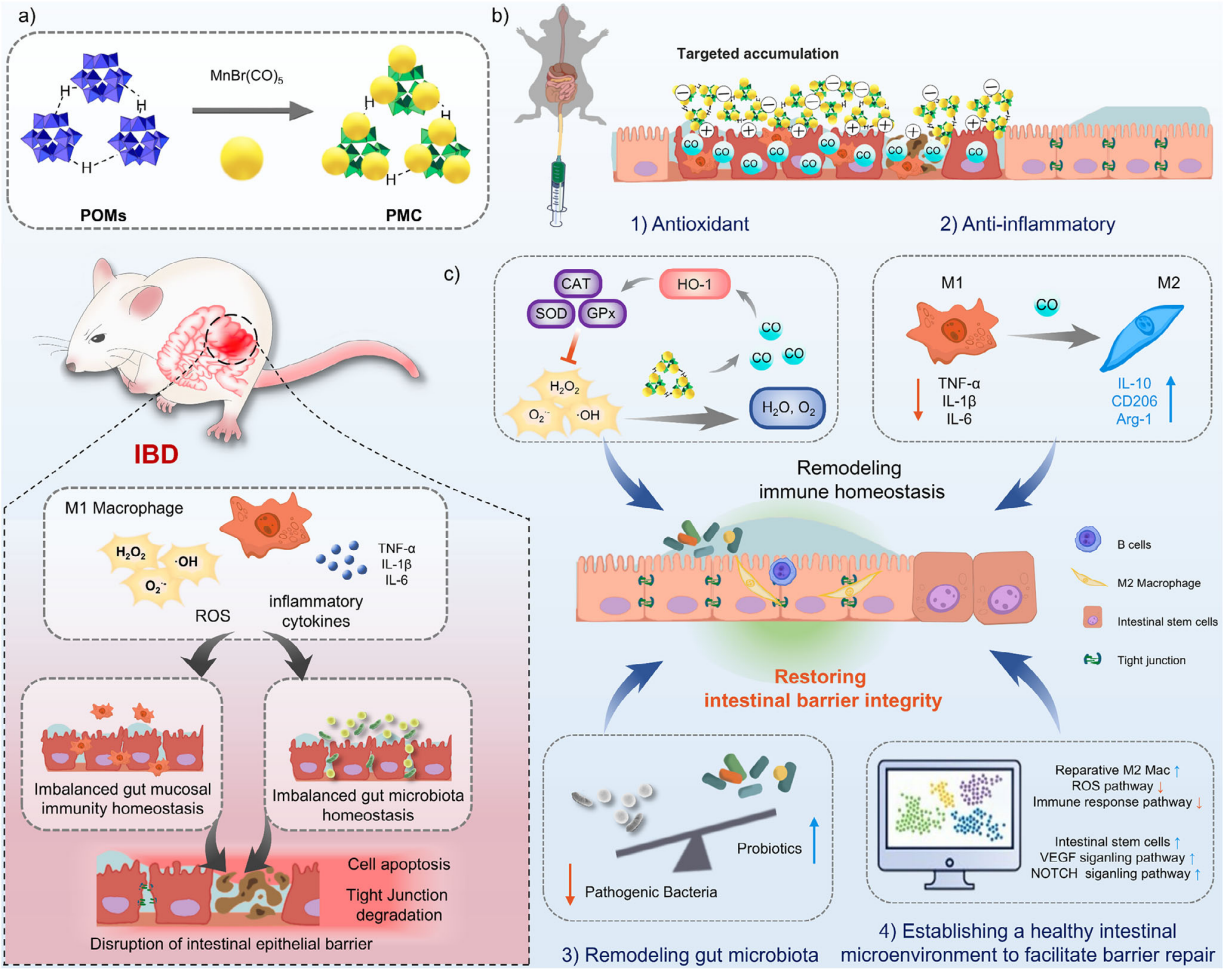
Schematic illustration of PMC preparation and its therapeutic mechanism for restoring intestinal barrier integrity through efficient remodeling of gut mucosal immunity and microbial homeostasis. 1) Antioxidant via scavenging ROS, activation of HO‐1, and enhancement of antioxidant enzyme activity. 2) Anti‐inflammatory via macrophage polarization toward the M2 phenotype and regulation of pro‐inflammatory and anti‐inflammatory cytokine expression. 3) Gut microbiota remodeling by suppressing harmful bacterial communities and enhancing beneficial bacterial abundance. 4) Establishing a healthy intestinal microenvironment to facilitate barrier repair.

Herein, we synthesized POMs utilizing ammonium molybdate and sodium dihydrogen phosphate, with ascorbic acid serving as the reducing agent. Subsequently, MnBr(CO)₅ was incorporated into the POMs via coordination interactions between Mn atoms in MnBr(CO)₅ and O atoms in the POMs structure, resulting in a CO‐releasing nanozyme, referred to as PMC (**Scheme** **1**a). By combining the advantageous properties of POMs and CO gas therapy, PMC demonstrated significant efficacy for ROS scavenging, anti‐inflammatory activity, and microbiota modulation. Following rectal administration, PMC effectively targeted the inflamed colonic mucosa via electrostatic interactions, scavenged ROS, and released CO in response to oxidative conditions, thereby exerting synergistic therapeutic effects (Scheme 1b). Both in vitro and in vivo experiments validated the exceptional antioxidant, anti‐inflammatory, and intestinal barrier‐repairing effects of PMC (Scheme 1c), with the therapeutic efficacy significantly surpassing that of POMs alone. 16S rRNA sequencing revealed that PMC significantly enhanced both the richness and diversity of the gut microbiota, effectively eliminating pathogenic bacteria at IBD sites. Single‐cell RNA sequencing (scRNA‐seq) further revealed that PMC reduced immune cell infiltration in the colons of mice with DSS‐induced colitis, while significantly increasing the proportion and number of colonic epithelial cells. Re‐clustering analysis demonstrated a notable decrease in the proportion of pro‐inflammatory M1 macrophages, with a significant suppression of inflammatory signaling pathways related to ROS and immune responses in macrophages. Additionally, the proportions of reparative M2 macrophages and intestinal stem cells increased significantly, in conjunction with prominent activation of the VEGF signaling pathway in macrophages and the NOTCH signaling pathway in intestinal stem cells. These results substantiate that PMC can effectively repair the intestinal barrier via robust antioxidant activity, suppression of inflammatory responses, activation of signaling pathways related to proliferation and repair, and promotion of intestinal stem cell proliferation and differentiation. This study demonstrates that PMC is a highly promising therapeutic agent for IBD, thereby providing novel insights and strategies for clinical treatment.

## 2 Results and Discussion

### 2.1 Preparation and Characterization of PMC

**Figure 1:**
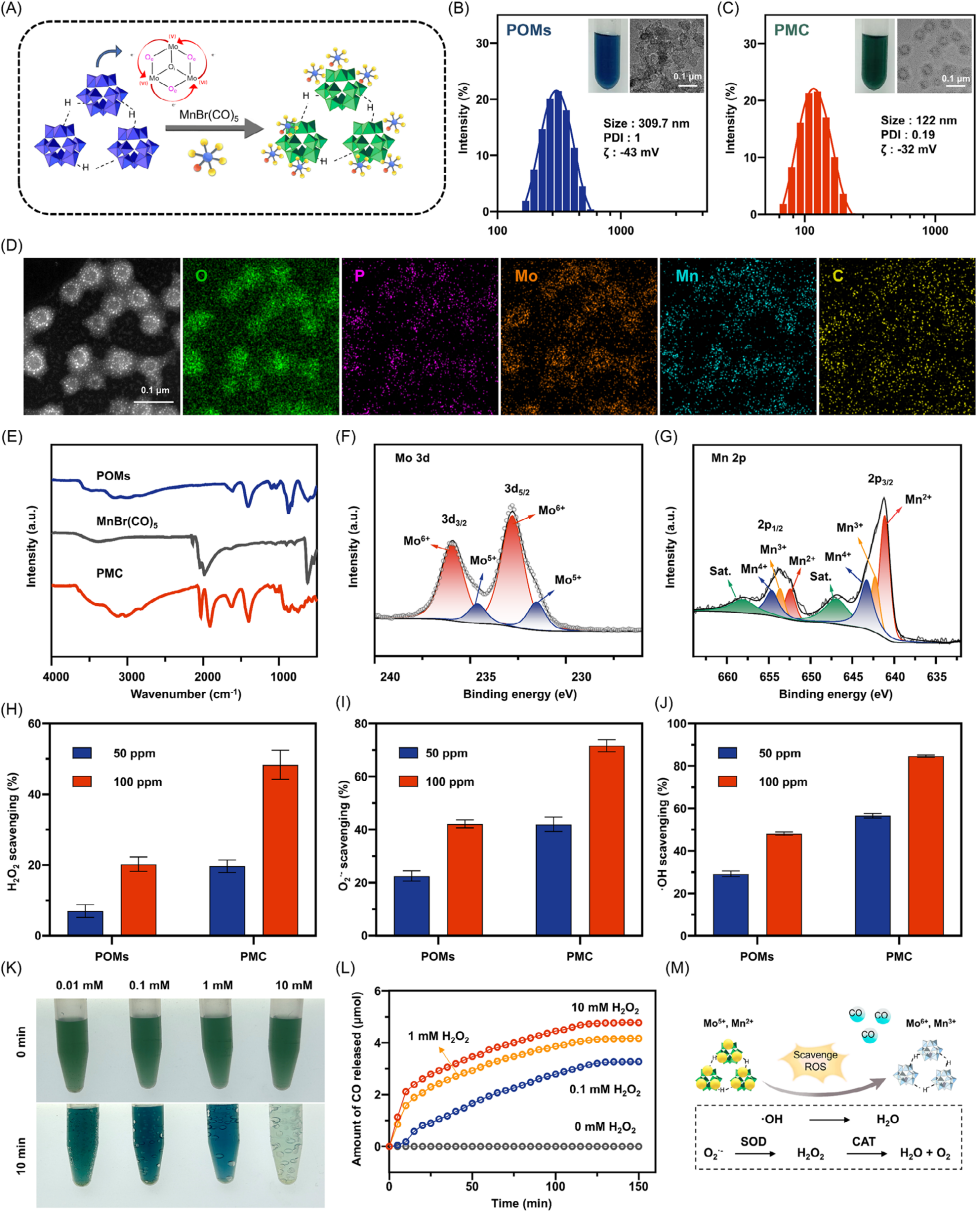
Physicochemical characterization of PMC nanoclusters. A) Schematic illustration of PMC preparation. Particle size distributions and TEM images of B) POMs and C) PMC at pH 5.5. D) Elemental mapping of PMC. E) FT‐IR spectra of POMs, MnBr(CO)₅, and PMC. XPS spectra of PMC for Mo 3*d* F) and Mn 2*p* G). ROS scavenging activities of H) H₂O₂, I) O₂·⁻, and J) ·OH by POMs and PMC. K, L) CO release profiles of PMC at different H₂O₂ concentrations. M) Proposed mechanism of ROS scavenging and CO release by PMC.

To synthesize the PMC nanozyme, a molybdenum‐based POMs cluster was initially prepared by dissolving ammonium molybdate, sodium dihydrogen phosphate, and ascorbic acid in an aqueous solution.^[^ ^30^ ^]^ This process yielded a clear blue solution due to the excellent solubility of POMs. The resulting POMs cluster consisted of Na⁺ and NH₄⁺ cations, integrated with Keggin‐type anionic structures. To functionalize the POMs with CO, MnBr(CO)₅ was added in methanol, allowing its coordination with the O atoms on POMs and resulting in the formation of the PMC (**Figure** **1**A). The resulting PMC solution had a clear green hue, indicating successful MnBr(CO)₅ modification. Characterization of PMC was performed using UV–visible (UV–vis) spectroscopy, where distinct peaks were observed at 380 and 870 nm, corresponding to MnBr(CO)₅ and POMs components, respectively, confirming the formation of PMC (Figure S1, Supporting Information). Particle size and morphology were further analyzed using dynamic light scattering (DLS) and transmission electron microscopy (TEM). Under conditions mimicking an inflamed environment (pH 5.5), as typically found in IBD, the average hydrodynamic diameter of POMs was measured to be 309.7 nm, with a polydispersity index (PDI) of 1 and a Zeta potential of −43 mV. TEM images revealed a hollow spherical structure for POMs, with particle sizes ranging from 10 to 300 nm (Figure 1B; Figure S2, Supporting Information). In contrast, the average hydrodynamic diameter of PMC decreased significantly to 122 ± 17.6 nm, with a PDI of 0.19 and a Zeta potential of −32 mV (Figure 1C). TEM mapping showed a uniform distribution of Mn and C atoms throughout the PMC, confirming the successful decoration of MnBr(CO)₅ onto the POMs (Figure 1D). To further investigate the interaction between POMs and MnBr(CO)₅, Fourier transform infrared (FTIR) spectroscopy and X‐ray photoelectron spectroscopy (XPS) were performed on POMs, MnBr(CO)₅, and PMC. The FTIR spectrum of the PMC revealed a notable blue shift in the carbonyl stretching vibration peak (1900–2100 cm⁻¹), likely due to the coordination interaction between the Mn atom in MnBr(CO)₅ and the O atoms in POMs, resulting in a reduction of electron density around the carbonyl group. Additionally, a red shift in the characteristic peak at 592 cm⁻¹, associated with the Mo─O─Mo bond, indicates an increase in electron density following coordination with Mn atoms (Figure 1E). A more detailed list of the important infrared peaks is shown in Table S1 (Supporting Information). XPS analysis revealed that the proportion of Mo⁵⁺ in POMs increased from 13.32% to 17.06% in PMC, while the proportion of Mn^2+^ increased from 13.47% in MnBr(CO)₅ to 47.91% in PMC (Figure 1F,G; Figure S3 and Table S2, Supporting Information). These results collectively suggest that the formation of the PMCs is driven primarily by coordination interactions between MnBr(CO)₅ and POMs.

POMs exhibit both catalase (CAT)‐like and superoxide dismutase (SOD)‐like activities, effectively scavenging various ROS through electron transfer between Mo⁵⁺ and Mo⁶⁺. In addition, in the presence of ROS, MnBr(CO)₅ undergoes an oxidation reaction that breaks the carbonyl ligand, leading to the release of CO. This process enables MnBr(CO)₅ to demonstrate a degree of ROS scavenging ability. This raises the question: how does the antioxidant performance of the PMC? To evaluate the efficacy of PMC in ROS scavenging, we assessed the scavenging effects of both PMC and POMs on three representative ROS: H₂O₂, O_2_^•−^, and ·OH. As shown in Figure 1H–J, the scavenging rates of POMs for H₂O₂, O_2_^•−^, and ·OH were 20.28%, 42.22%, and 48.18%, respectively. In contrast, the scavenging rates of PMC at the same Mo ions concentration were significantly higher, reaching 48.38%, 71.69%, and 84.73%, respectively. This indicates that PMC exhibits superior ROS scavenging performance compared to POMs, primarily due to the increased ratio of Mo^5+^ and Mn^2+^. Previous studies have shown that Mn^2+^ possesses excellent CAT‐like and SOD‐like activities,^[^ ^31^ ^]^ meaning the high Mn^2+^ content in PMC significantly enhances its antioxidant capacity. Notably, the enhanced antioxidant activity of PMC may also be related to its increased stability. In a weakly acidic environment, hydrogen bonding interactions between H^+^ ions in the solution and the O atoms of POMs enhance the attraction between adjacent anions, while significantly reducing electrostatic repulsion. This leads to the self‐assembly of POMs into larger hollow aggregates (as evidenced by the particle size of POMs being only 5 nm at pH 7.4, Figure S4, Supporting information), which can reduce the antioxidant activity of POMs. However, after coordination with MnBr(CO)₅, the coordination between MnBr(CO)₅ and the O atoms of POMs partially inhibits hydrogen bond formation with H^+^ ions, preventing further aggregation and significantly improving the stability of POMs. The enhanced stability of PMC in the weakly acidic environment typical of IBD is expected to further boost its antioxidant effects, thereby increasing its potential for application in IBD treatment.

PMC not only scavenges ROS but also responsively releases CO, as demonstrated in Figure 1K. The results show that as the concentration of H₂O₂ increases from 0.01 to 10 mm, both the amount and the rate of CO release significantly rise. This indicates that PMC exhibits a high responsiveness to H₂O₂, facilitating the release of CO in ROS‐rich inflammatory lesions. Additionally, we used a CO detector to monitor the real‐time CO release behavior of PMC. After incubating PMC in 1 and 10 mm H₂O₂ for 2.5 h, the cumulative CO release amounts were 4.16 and 4.77 µmol, respectively (Figure 1L). These findings suggest that PMC not only exerts efficient antioxidant effects at the IBD site but also enables controllable CO release (Figure 1M), offering a potential multi‐faceted therapeutic mechanism for anti‐inflammatory effects and the treatment of IBD. PMC demonstrates good biocompatibility. As shown in Figure S5A–C (Supporting Information), the cell viability of Raw264.7 and Caco‐2 cells remained above 85% at Mo ion concentrations below 200 µg mL^−1^, with negligible propidium iodide (PI)‐labeled dead cells observed. Additionally, the hemolysis rates for both POMs and PMC were below 5%, as indicated in Figure S6 (Supporting Information).

### 2.2 PMC's In Vitro Antioxidant and Anti‐Inflammatory Effects

**Figure 2:**
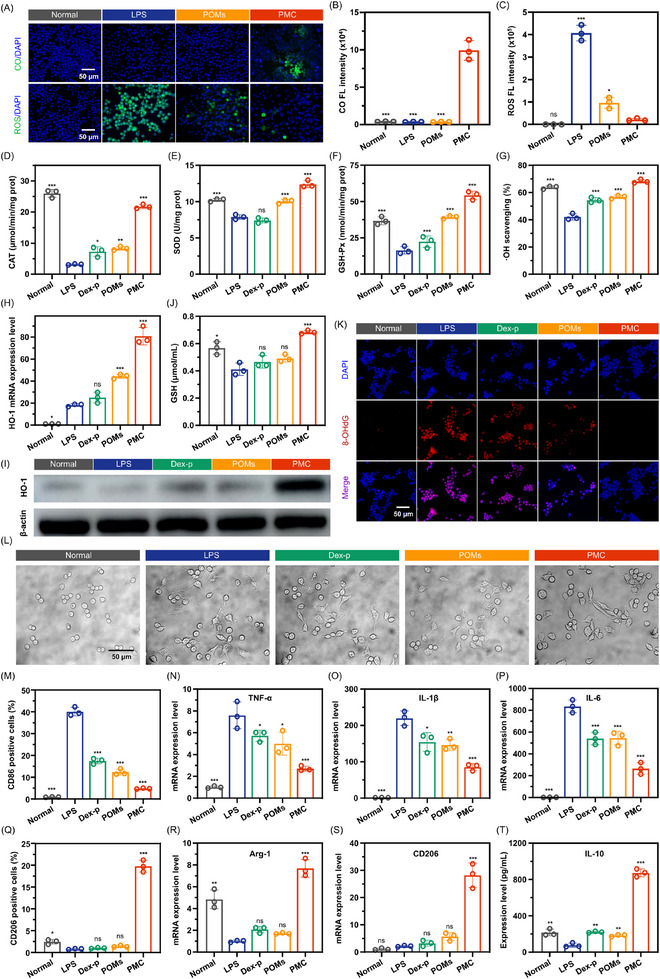
In vitro antioxidant and anti‐inflammatory activities of PMC. A–C) Intracellular CO imaging and ROS scavenging, and corresponding fluorescence intensity. Enzymatic activity of D) CAT, E) SOD, and F) GSH‐Px, along with G) ·OH scavenging capacity in PMC‐treated activated macrophages. H, I) mRNA and protein expression levels of HO‐1 in the PMC group. J) GSH content in the PMC group. K) Immunofluorescence staining of 8‐OHdG‐positive cells in the PMC group. L) Morphological changes in RAW 264.7 macrophages after various treatments. M, Q) Flow cytometry analysis of macrophage phenotype following various treatments. mRNA expression levels of pro‐inflammatory cytokines: N) TNF‐α, O) IL‐1β, and P) IL‐6, along with M2 phenotype markers R) Arg‐1 and S) CD206. T) IL‐10 level in PMC‐treated activated macrophages. All statistical analyses were compared by comparing the LPS group with other groups (n = 3), with ^*^ *p* < 0.05, ^**^ *p* < 0.01, ^***^ *p* < 0.001, and ns indicating no significant difference.

Before assessing the antioxidant activity of PMC, we first investigated its CO release behavior in LPS‐induced activated macrophages. Using a CO probe system (PdCl₂ + FL‐CO‐1), which emits green fluorescence only in the presence of CO, we observed that PMC rapidly generated a significant amount of CO in activated macrophages, resulting in pronounced green fluorescence, as shown in **Figure** **2**A,B. Concurrently, PMC effectively scavenged intracellular ROS, as evidenced by the dichlorofluorescein (DCF) and H_2_O_2_ fluorescence assays, which showed almost no green fluorescence in PMC‐treated cells (Figure 2A,C; Figure S7, Supporting Information). POMs also demonstrated ROS scavenging capability, as the DCF and H_2_O_2_ fluorescence intensities in POMs‐treated cells was markedly lower than that in the LPS group. After confirming that PMC could release CO intracellularly and scavenge ROS, we next examined its impact on the activities of intracellular antioxidant enzymes. As shown in Figure 2D–G, the antioxidant enzyme activities in activated macrophages were significantly lower compared to those in normal macrophages. Dexamethasone Sodium (Dex) slightly increased the antioxidant enzyme activity. In contrast, the activities of CAT, SOD, and GSH‐Px were notably increased by 2.62, 1.27, and 2.43 times, respectively, after treatment with POMs, with an ·OH scavenging rate of up to 56.76%. These results indicate that POMs possesses good antioxidant activity. Remarkably, CO further significantly boosted the antioxidant activity of POMs. In PMC‐treated cells, the activities of CAT, SOD, and GSH‐Px reached or even exceeded those of normal macrophages, with an ·OH scavenging rate of up to 69.76%, significantly higher than that of the normal group (63.77%). Further research revealed that the enhanced antioxidant activity of PMC is primarily related to the activation of intracellular HO‐1 expression. HO‐1 is a crucial antioxidant defense enzyme that prevents oxidative damage to intracellular biomolecules (such as proteins, lipids, and DNA) by catalyzing the degradation of heme, producing bilirubin, iron ions, and carbon monoxide (CO), and regulating the activity of antioxidant enzymes. The mRNA (Figure 2H) and protein expression levels (Figure 2I; Figure S8, Supporting Information) of HO‐1 in PMC‐treated activated macrophages were significantly higher than in other treatment groups, being 4.46 and 13.43 times greater than in the LPS group, respectively. Additionally, PMC treatment significantly upregulated intracellular GSH levels (Figure 2J). The increase in GSH content can reduce Mo^6+^ to the antioxidant Mo^5+^, enabling PMC to exert a lasting ROS scavenging effect. In summary, PMC can rapidly release large amounts of CO in activated macrophages, and the generated CO significantly enhances the antioxidant activity of POMs by activating HO‐1 and increasing GSH expression. Furthermore, the substantial scavenging of ROS and the marked increase in various antioxidant enzyme activities led to a significant reduction in ROS‐induced DNA damage. As shown in Figure 2K; Figure S9 (Supporting Information), in LPS‐treated activated macrophages, 8‐OHdG (a common biomarker of DNA damage due to oxidative stress) exhibited strong red fluorescence, whereas in PMC‐treated activated macrophages, this fluorescence was almost undetectable, highlighting the significant role of CO in scavenging ROS and mitigating DNA oxidative damage.

After confirming the potent antioxidant activity of PMC, we further evaluated its anti‐inflammatory effects. As shown in Figure 2L, LPS‐treated activated macrophages displayed a dendritic morphology with numerous pseudopodia, characteristic of M1 macrophages. In contrast, following PMC treatment, these macrophages exhibited a more elongated morphology with significantly reduced pseudopodia. This elongated shape increases the surface area of the cell membrane, facilitating more effective secretion of anti‐inflammatory cytokines and growth factors by M2 macrophages, thereby promoting intercellular signaling and tissue healing. To further confirm the effect of PMC on M2 macrophage polarization, we performed fluorescence labeling of the treated macrophages using CD86 (an M1 marker) and CD206 (an M2 marker) and analyzed the changes via flow cytometry. The results showed that the proportion of CD86‐labeled M1 macrophages in the LPS group was 39.4%, which decreased slightly to 16.0% and 11.4% in the Dex‐p and POMs groups, respectively (Figure 2M; Figure S10, Supporting Information). In contrast, PMC treatment significantly reduced the proportion of M1 macrophages to 4.72%. Furthermore, the proportion of M2 macrophages significantly increased to 19.7% following PMC treatment, compared to only 0.65% in the LPS group (Figure 2Q; Figure S10, Supporting Information). These findings indicate that PMC treatment significantly promotes the polarization of macrophages toward the M2 phenotype. PCR analysis confirmed that PMC treatment significantly downregulated the expression of pro‐inflammatory cytokines TNF‐α, IL‐1β, IL‐6, and iNOS, with reductions of 2.83, 2.60, 3.25, and 4.47 times compared to the LPS group, respectively (Figure 2N–P; Figure S11C, Supporting Information). Correspondingly, protein expression levels of TNF‐α and IL‐6 also showed significant reductions, decreasing by 2.95 and 3.19 fold, respectively, after PMC treatment compared to the LPS group (Figure S11A,B, Supporting Information). Additionally, PMC treatment markedly upregulated the expression of M2 macrophage markers Arg‐1 and CD206, which increased by 7.78 and 13.28 fold, respectively, compared to the LPS group (Figure 2R,S). ELISA results further highlighted the potent anti‐inflammatory effect of PMC, showing that IL‐10 levels in the LPS group were only 76.48 pg/mL, whereas PMC treatment elevated the concentration to 873.49 pg mL^─1^—an 11.4‐fold increase (Figure 2T). In summary, PMC significantly reduces the proportion of M1 macrophages and inhibits the release of pro‐inflammatory cytokines, promoting the polarization of macrophages toward M2 phenotype. Additionally, PMC exhibits significant anti‐inflammatory effects by increasing IL‐10 release. These findings underscore the potential application of PMC in the treatment of IBD.

### 2.3 In Vitro Reparative Effects of PMC on the Intestinal Barrier

**Figure 3:**
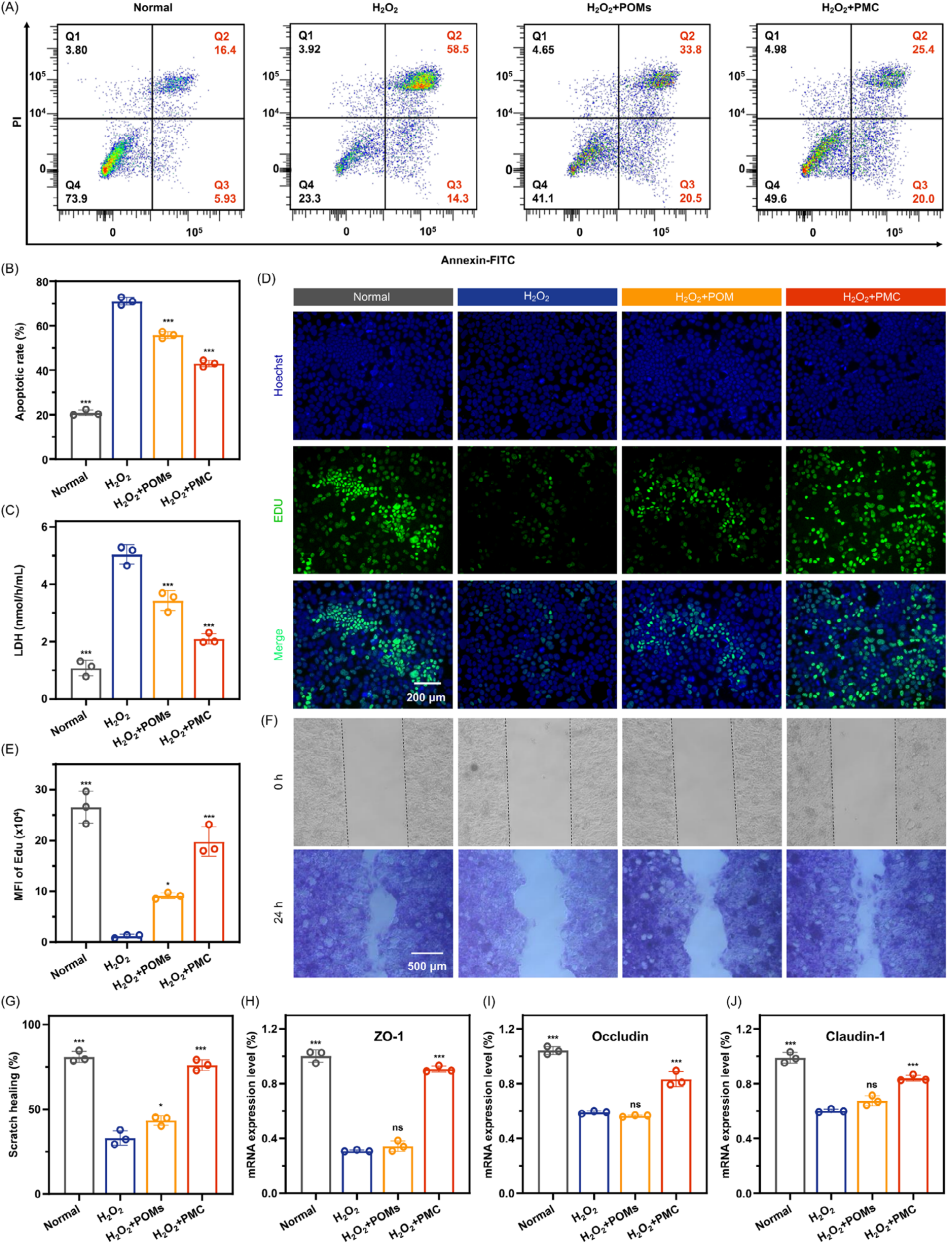
Effect of PMC on intestinal epithelial barrier repair. A, B) Flow cytometry analysis of Caco‐2 cell apoptosis following various treatments. C) LDH content in the supernatant of Caco‐2 cells after treatment with different conditions. D, E) EdU staining and (F, G) scratch‐healing assays of Caco‐2 cells after various treatments. mRNA expression levels of tight junction proteins: H) ZO‐1, I) Occludin, and J) Claudin‐1 after different treatments. All statistical analyses were performed by comparing the H₂O₂ group with other groups (n = 3), with ^*^ *p* < 0.05, ^***^ *p* < 0.001, and ns indicating no significant difference.

The disruption of the intestinal mechanical barrier is primarily caused by apoptosis of intestinal epithelial cells and the breakdown of tight junctions, both of which are induced by ROS and pro‐inflammatory cytokines. Given the superior antioxidant and anti‐inflammatory properties of PMC observed in vitro, we further explored its potential in repairing this critical barrier. In this study, we used a H₂O₂‐induced Caco‐2 cell oxidative damage model to systematically evaluate the effects of PMC on inhibiting cell apoptosis, promoting cell proliferation, accelerating cell scratch healing, and enhancing tight junction protein expression. As shown in Figure S12 (Supporting information), treatment with 1 mM H₂O₂ caused significant cell death in Caco‐2 cells, with a large number of dead cells marked by PI in the H₂O₂ group, resulting in a cell viability of only 63.3%. In contrast, in the PMC group, only a small number of PI‐labeled dead cells were observed, and cell viability increased to 101.2%. Further analysis revealed that H₂O₂‐induced oxidative damage increased the apoptosis rate of Caco‐2 cells to 72.8% (**Figure** **3**A,B), while the apoptosis rate in the PMC group decreased to 45.4%. These results suggest that PMC effectively scavenges H₂O₂, thereby inhibiting H₂O₂‐induced apoptosis in Caco‐2 cells. Since apoptosis increases membrane permeability, we further evaluated the protective effect of PMC on H₂O₂‐induced apoptosis by measuring lactate dehydrogenase (LDH) levels in the supernatant, which serves as an indicator of membrane damage. As shown in Figure 3C, after H₂O₂ treatment, the LDH levels in the supernatant significantly increased, indicating severe membrane damage. However, in the PMC group, the LDH levels decreased by 2.5 times compared to the H₂O₂ group, further confirming that PMC effectively protects Caco‐2 cells from H₂O₂‐induced apoptosis. In addition to inhibiting apoptosis, PMC significantly promoted the proliferation and scratch healing abilities of Caco‐2 cells. As shown in Figure 3D,E and H₂O₂ treatment markedly reduced Caco‐2 cell proliferation, as evidenced by a significant decrease in EdU‐positive cells compared to the normal group. However, after treatment with POMs and PMC, especially in the PMC group, the number of EdU‐positive cells significantly increased, nearly returning to normal levels. Fluorescence intensity analysis confirmed this trend, demonstrating that PMC promotes Caco‐2 cell proliferation. Concurrently, PMC treatment significantly improved the scratch healing ability of Caco‐2 cells. As shown in Figure 3F,G, after 24 h of co‐culturing with PMC, the width of the Caco‐2 cell scratch decreased from an initial value of 0.99 to 0.22 mm, achieving a healing rate of 76.10%. This rate was significantly higher than the healing rates of the H₂O₂ and POMs groups (33.09% and 43.50%, respectively), and comparable to the healing rate of the normal group (80.93%). On top of protecting Caco‐2 cells from ROS‐induced apoptosis and promoting cell proliferation and scratch healing, PMC also significantly increased the expression levels of tight junction proteins, including ZO‐1, Occludin, and Claudin‐1, in Caco‐2 cells. Compared to the H₂O₂ group, the mRNA expression levels of these proteins were upregulated by 2.92, 1.41, and 1.39 times, respectively, in the PMC group (Figure 3H–J). The significant upregulation of these tight junction proteins indicates that PMC has considerable potential in repairing cell membrane structure and restoring tight junctions, providing strong support for protecting against ROS‐induced intestinal barrier damage.

### 2.4 Therapeutic Effect of PMC in the DSS‐Induced IBD Mouse Model

**Figure 4:**
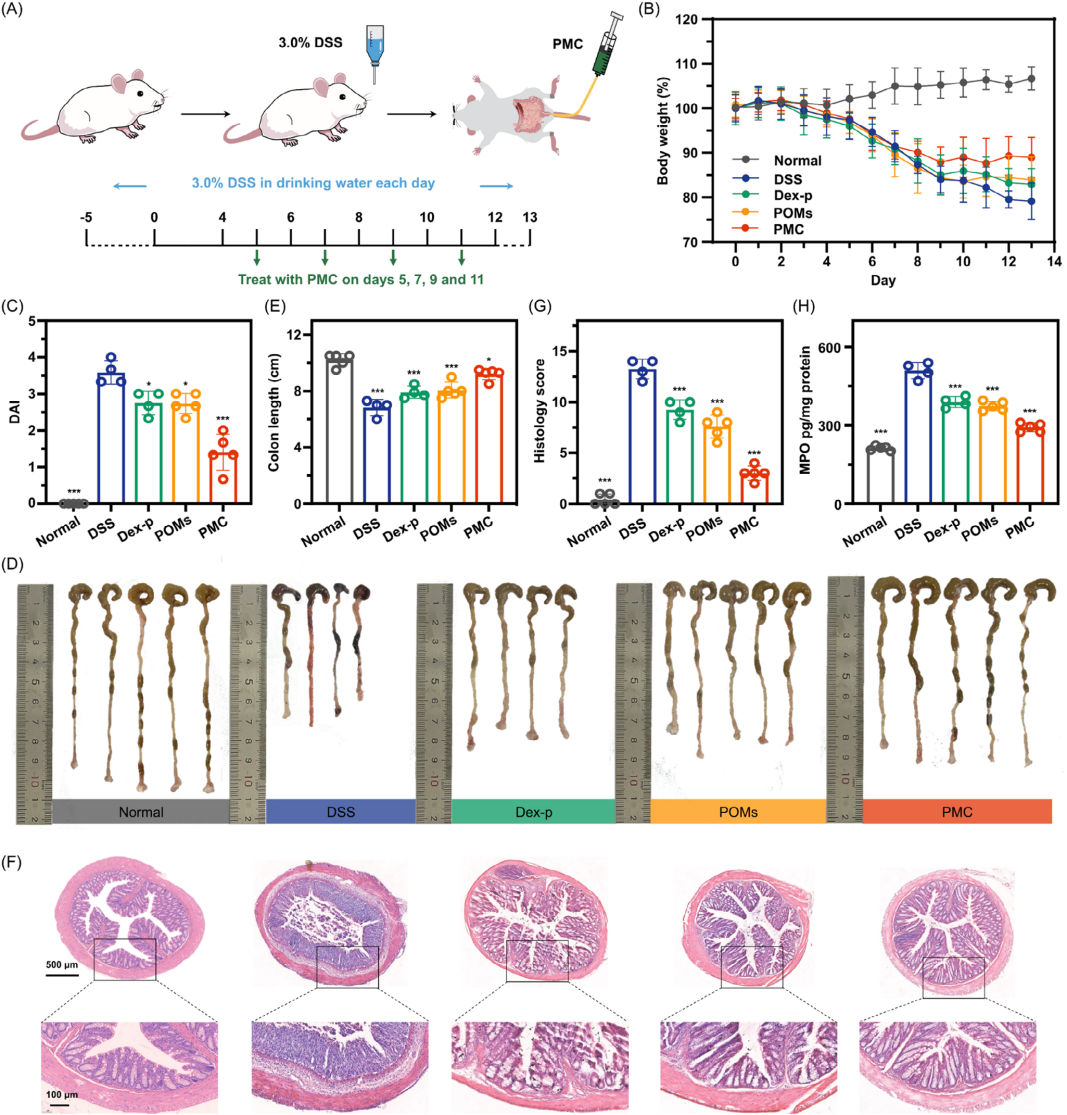
Therapeutic efficacy of PMC in a mouse model of DSS‐induced colitis. A) Experimental design of the DSS‐induced IBD mouse model. B) Daily body weight changes over 13 days. C) DAI index. D, E) Colon length of mice following the indicated treatments at the end of the study. F, G) Representative H&E staining images and histology scores of colon tissue after the indicated treatments; H) MPO activity in colon tissues from each group. Statistical analyses were performed by comparing the DSS group with other groups (n = 5). ^*^ *p* < 0.05, ^***^ *p* < 0.001.

All animal experiments in this study were approved and supervised by the Animal Ethics Committee of Wenzhou Medical University (approval number: wydw 2024‐0337). Given the remarkable antioxidant, anti‐inflammatory, and intestinal barrier repair properties of PMC observed in vitro, we further investigated its therapeutic efficacy in a DSSinduced UC colitis mouse model (**Figure** **4**A). Before systematically evaluating its therapeutic effects, we first assessed the targeted accumulation and retention behavior of PMC at the inflamed colon sites. As expected, PMC demonstrated excellent IBD targeting, with significantly higher Mo and Mn content in the inflamed colon regions compared to healthy controls at both 6 and 12 h post‐retecal administration (Table S4, Supporting Information). This targeted retention is primarily attributed to the strong electrostatic interactions between the negatively charged PMC and the positively charged glycoproteins on the damaged mucosal surface. Body weight is a critical indicator for evaluating therapeutic effects in colitis. As shown in Figure 4B, throughout the treatment period, body weight in most colitis mice consistently decreased. Notably, the DSS group exhibited the most significant weight loss, with a decrease of 20.86% on day 13 In contrast, weight loss in the Dex‐p and POMs groups was more modest, at 17.08% and 16.04%, respectively. The PMC group demonstrated the least weight reduction, with only a 11.01% decrease. Moreover, PMC treatment significantly alleviated key symptoms of colitis, including rectal bleeding and diarrhea. The Disease Activity Index (DAI), a quantitative measure of disease severity and treatment efficacy based on stool consistency, rectal bleeding, and body weight changes, revealed important findings. A higher DAI score indicates more severe inflammation. Not surprisingly, the DSS group exhibited the highest DAI score of 3.5, reflecting severe disease. The Dex‐p and POMs groups showed partial symptom alleviation, with DAI scores of 2.8 and 2.7, respectively. In contrast, the PMC group exhibited the lowest DAI score of 1.4 (Figure 4C), suggesting substantial therapeutic efficacy. To further assess PMC's therapeutic potential, we measured the excised colon length on day 13. Chronic inflammation often leads to smooth muscle contraction and mucosal damage, resulting in reduced colon length. As shown in Figure 4D,E, the average colon length in healthy mice was 10.2 cm, while the colon lengths in the DSS, Dex‐p, and POMs groups were reduced by 33.09%, 22.55%, and 20.98%, respectively. The PMC group exhibited the least reduction, with colon length decreasing by only 9.80%. Histological analysis of colon tissues using hematoxylin and eosin (H&E) staining revealed extensive damage in DSS‐induced colitis. The DSS group exhibited widespread crypt loss and inflammatory cell infiltration, with a histological score of 12.6 (Figure 4F,G). In contrast, the colon tissue of PMC group showed significant improvements, including reduced goblet cell loss and less inflammatory cell infiltration around the crypts, resulting in a significantly lower histological score of 3.0. Additionally, PMC treatment notably reduced myeloperoxidase (MPO) levels, a marker of neutrophil infiltration, further supporting its anti‐inflammatory effect. The MPO levels in the PMC group were reduced to 291.37 pg mL^−1^, significantly lower than the DSS (509.07 pg mL^−1^), Dex‐p (388.03 pg mL^−1^), and POMs groups (373.37 pg mL^−1^) (Figure 4H), suggesting that PMC effectively inhibits neutrophil recruitment to the inflamed tissue. PMC treatment showed excellent biocompatibility, as evidenced by minimal occurrence of occult blood in treated mice (Figure S13, Supporting Information) and no adverse effects on hematological parameters. The red blood cell count, white blood cell count, and HbCO levels (a key marker of CO poisoning) in colitis mice treated with PMC were nearly identical to those of healthy mice (Figure S14A–C, Supporting Information). No significant damage to vital organs was observed, further supporting the safety and biocompatibility of PMC (Figure S15, Supporting Information). In conclusion, the results from this study strongly support PMC's therapeutic potential in the treatment of ulcerative colitis.

**Figure 5:**
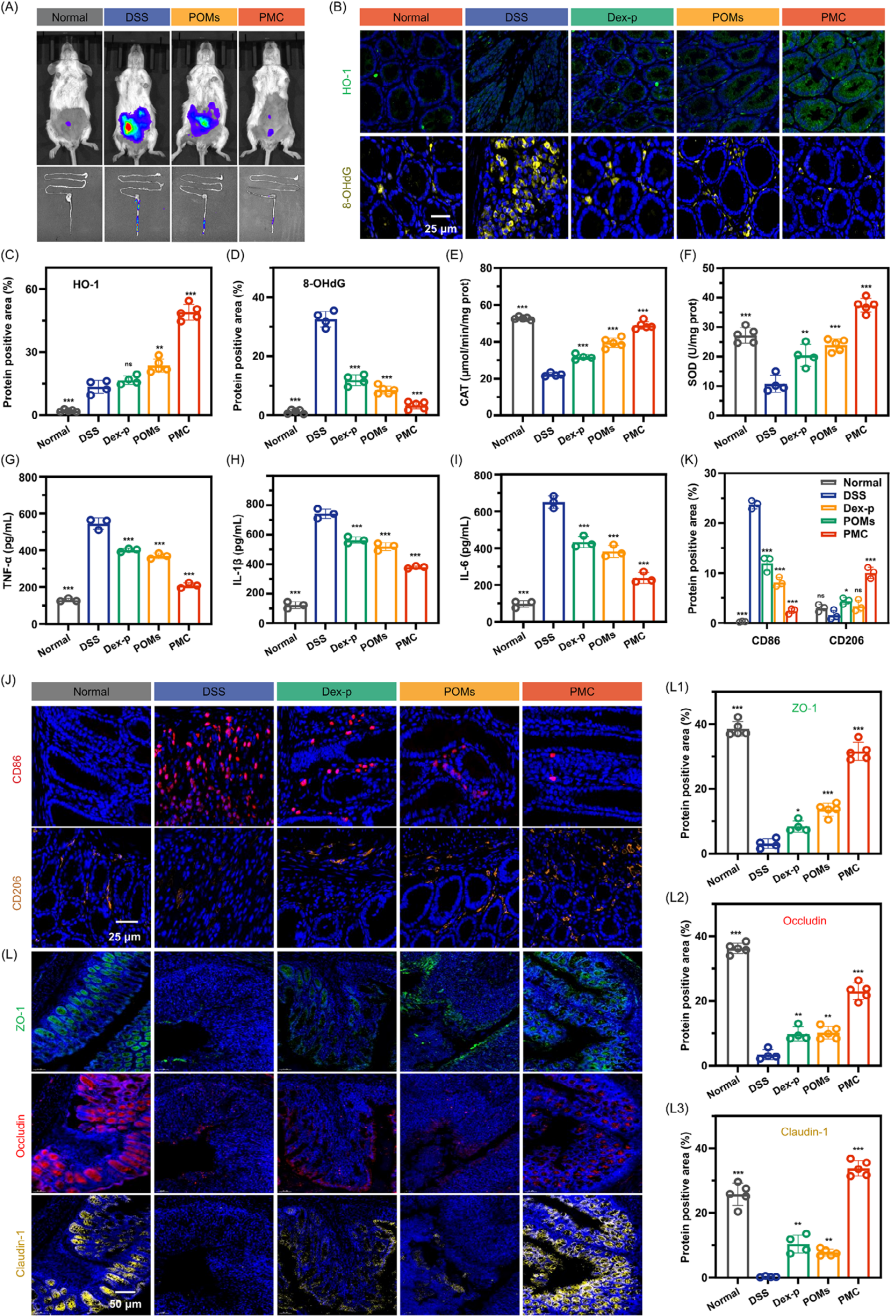
In Vivo antioxidant, anti‐inflammatory, and intestinal epithelial barrier repair effects of PMC. A) Fluorescence images showing in vivo and ex vivo ROS scavenging activity of PMC. B–D) Expression levels of HO‐1 and 8‐OHdG in colonic tissue. Enzymatic activity of E) CAT and F) SOD in colonic tissue. Pro‐inflammatory cytokine expression levels in colonic tissue: G) TNF‐α, H) IL‐1β, and I) IL‐6. J, K) CD86‐positive and CD206‐positive cells in colonic tissue. L) Expression levels of tight junction proteins in colonic tissue. Statistical analyses were performed by comparing the DSS group with other groups (n = 5), with ^*^ *p* < 0.05, ^**^ *p* < 0.01, ^***^ *p* < 0.001, and ns indicating no significant difference.

To further confirm the therapeutic efficacy of PMC in vivo, we evaluated its ability to eliminate ROS, suppress inflammation, promote intestinal barrier repair, and regulate gut microbiota at the site of IBD. ROS levels were measured using the L012 chemiluminescent probe, which is commonly used to monitor ROS in inflammatory diseases. In the DSS‐induced colitis mouse model, intense fluorescence signals were observed, particularly in the colon, indicating elevated ROS levels. After treatment with POMs and PMC, these fluorescence signals were significantly reduced, with the PMC group showing nearly identical fluorescence intensity to the healthy control group, suggesting a substantial clearance of ROS at the site of inflammation (**Figure** **5**A). Additionally, PMC treatment significantly increased the expression of HO‐1 in colon tissue (Figure 5B,C) and alleviated oxidative DNA damage, as evidenced by the marked reduction in 8‐OHdG expression (Figure 5B,D). Additional analysis revealed that PMC significantly enhanced the activity of key antioxidant enzymes, including CAT and SOD in colon tissue (Figure 5E,F). These enzyme activities were significantly higher than those in the Dex‐p and POMs groups, further demonstrating PMC's potent antioxidant effect. Beyond its antioxidant effects, CO also plays a pivotal role in modulating inflammation, as evidenced by two key observations. 1) In the DSS‐induced colitis model, the DSS group exhibited significantly elevated levels of pro‐inflammatory cytokines (TNF‐α: 544.3 pg mL^−1^, IL‐1β: 743.2 pg mL^−1^, IL‐6: 650.2 pg mL^−1^). In contrast, the PMC group showed markedly reduced cytokine levels (TNF‐α: 209.7 pg mL^−1^, IL‐1β: 379.6 pg mL^−1^, IL‐6: 238.6 pg mL^−1^), which were comparable to the levels observed in healthy mice (TNF‐α: 129.1 pg mL^−1^, IL‐1β: 122.1 pg mL^−1^, IL‐6: 97.7 pg mL^−1^) (Figure 5G–I). 2) Immunofluorescence analysis revealed that PMC treatment inhibited the differentiation of M1 macrophages (CD86‐positive) while promoting the differentiation of M2 macrophages (CD206‐positive) in colon tissue (Figure 5J,K). These findings suggest that PMC effectively shifts macrophage polarization from the pro‐inflammatory M1 phenotype to the anti‐inflammatory M2 phenotype, thereby alleviating excessive inflammation and facilitating tissue repair. The integrity of the intestinal barrier is crucial in maintaining gut homeostasis, and tight junction proteins, such as ZO‐1, Occludin, and Claudin‐1, are essential for intestinal epithelial barrier function. In the DSS model, the expression of these proteins was nearly undetectable, indicating significant degradation of these proteins. In contrast, the PMC group showed strong immunofluorescent signals for ZO‐1, Occludin, and Claudin‐1, suggesting that PMC treatment significantly prevented the degradation of these proteins and protected the intestinal barrier (Figure 5L1‐3). To further assess the impact of PMC on intestinal permeability, we used FITC‐labeled dextran (FITC‐Dex, Mw = 4 kDa) as a fluorescent probe. FITC‐Dex cannot cross an intact intestinal barrier, but when the barrier is compromised, it enters the bloodstream. Not surprisingly, the DSS group showed the highest fluorescence intensity of FITC‐Dex in the blood supernatant, indicating increased intestinal permeability. In contrast, the PMC group exhibited a significant reduction in fluorescence intensity, with levels nearly identical to those in the healthy control group, suggesting that PMC effectively restored intestinal barrier function and reduced permeability (Figure S16, Supporting Information). Together, the results of this study provide compelling evidence for the therapeutic potential of PMC in treating IBD. PMC effectively reduced ROS levels, suppressed inflammation, promoted intestinal barrier repair, making it a promising candidate for IBD therapy.

**Figure 6:**
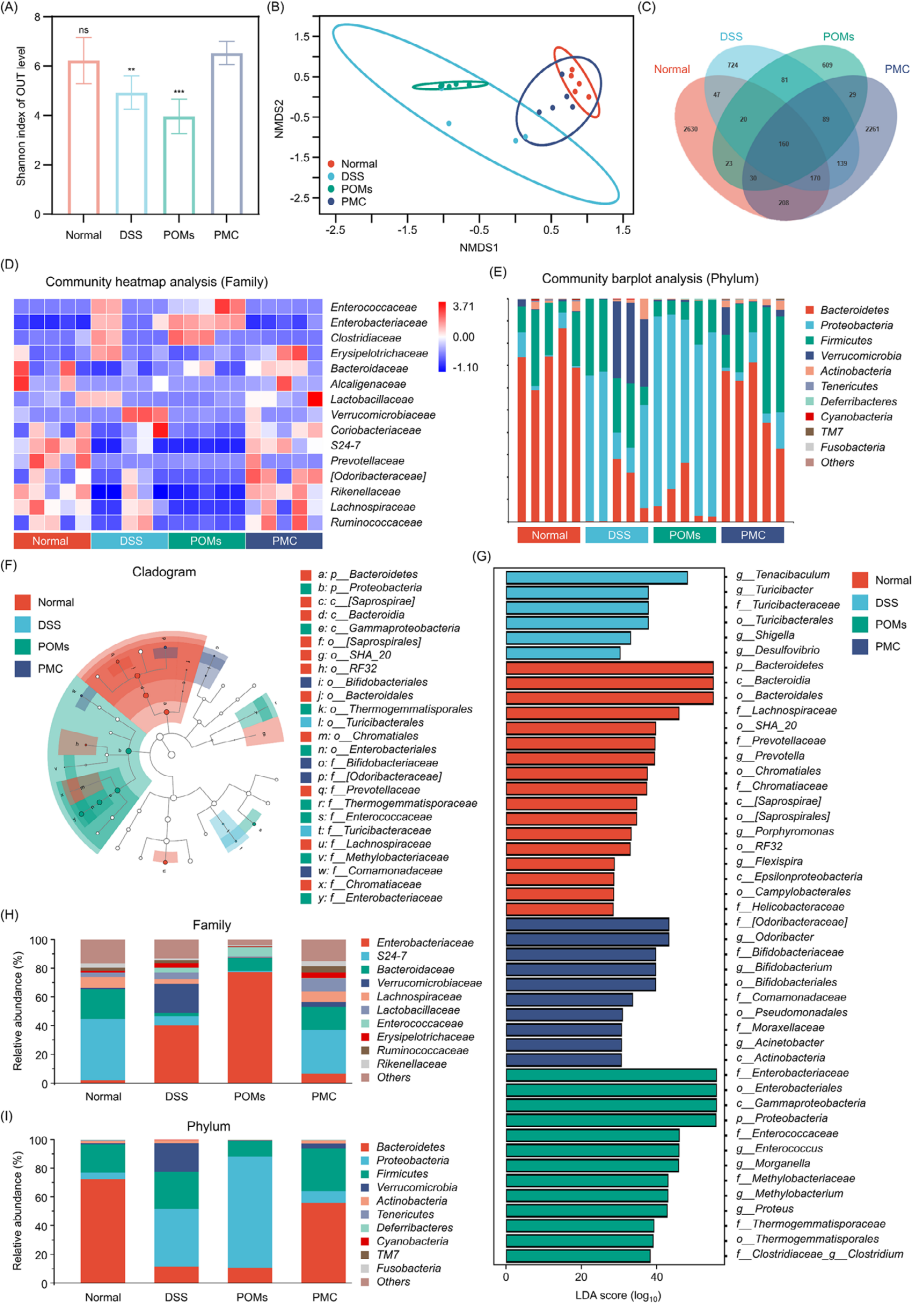
Effect of PMC on gut microbiome structure and composition. A) *α*‐diversity analysis using the Shannon index (n = 5). B) NMDS analysis, where each point represents a sample. C) Venn diagram illustrating the differences in microbiota composition between different treatment groups. D) Heatmap showing the relative abundances of microbial profiles at the family level, with each column representing one mouse. E) Community bar plot depicting microbial composition at the phylum level, with each row representing one mouse. F) Cladogram showing the differences in richness and identifying groups with significantly different abundance. The brightness of each dot is proportional to its effect size. G) LDA identifying significantly abundant genera in different groups. H, I) Relative abundance of microbiota significantly altered at the family and phylum levels.

When the intestinal barrier is compromised, substantial numbers of pathogenic bacteria can infiltrate the intestinal lumen. Once activated by these pathogens, innate immune cells initiate a cascade of inflammatory responses that can exacerbate IBD. Therefore, maintaining a balanced gut microbiota is essential for sustaining intestinal health. Given the demonstrated in vivo antioxidant, anti‐inflammatory, and intestinal barrier repair properties of PMC, we employed 16S ribosomal RNA (rRNA) gene sequencing to explore its regulatory effects on the gut microbiota. Significant alterations in gut microbiota were observed in DSS‐treated mice. The Shannon indices shown in **Figure** **6**A illustrate a significant reduction in gut microbiota diversity in the DSS group. Treatment with PMC, however, substantially improved these indices, aligning them more closely with those of the healthy control group. Non‐metric Multidimensional Scaling (NMDS) analysis further supported these findings by demonstrating that the gut microbiota composition in the PMC group was similar to that of the healthy control group, while the DSS group exhibited a distinct composition, validated by a stress value of 0.05 (Figure 6B). This finding was further validated by Venn diagram analysis (Figure 6C). The microbial composition at both family and phylum levels for the Normal, DSS, POMs, and PMC groups was illustrated through heatmaps and bar graphs (Figure 6D,E). Differentially abundant taxa across the groups were identified using Linear Discriminant Analysis Effect Size (LEfSe) and Linear discriminant analysis (LDA) scoring, highlighting dominant taxa and their variations from phylum to genus (Figure 6F,G). The composition analysis revealed that PMC treatment notably altered the gut microbiota compared to the DSS group (Figure 6H,I). Key findings include: 1) PMC treatment notably enhanced the relative abundance of beneficial bacteria, particularly within the Bacteroidetes phylum, such as Bacteroidaceae, Ruminococcaceae, and S24‐7.^[^ ^32^ ^]^ The increased presence of Lachnospiraceae indicates that PMC effectively suppressed intestinal inflammation.^[^ ^33^ ^]^ 2) PMC treatment reduced the relative abundance of potentially harmful bacteria, particularly Enterobacteriaceae and Proteobacteria,^[^ ^34^ ^]^ known to thrive in inflamed intestines. Reducing these bacteria is essential for mitigating IBD. The bacterial classification of important pathogenic and beneficial bacteria at the family and phylum levels is listed in Table S5 (Supporting Information). 3) PMC restored the microbial balance, aligning the relative abundance of both beneficial and harmful bacteria closely with that of the healthy control group, signifying effective regulation of the gut microbiota and promotion of beneficial bacterial growth. Although POMs were able to partially alleviate oxidative stress in IBD‐affected regions, their influence on gut microbiota diversity and richness was significantly less pronounced compared to PMC. This observation suggests that POMs have limited regulatory effects,^[^ ^13a^ ^]^ with CO playing a primary role in microbiota modulation. Summarized, these results show that PMC effectively increases gut microbiota diversity and richness, reduces pathogenic bacteria, and promotes beneficial bacterial growth. This restructuring of the gut microenvironment is crucial for microbiota balance and significantly improves colitis treatment outcomes.

### 2.5 scRNA‐Seq Reveals the Underlying Mechanisms of PMC to Promote IBD Recovery

**Figure 7:**
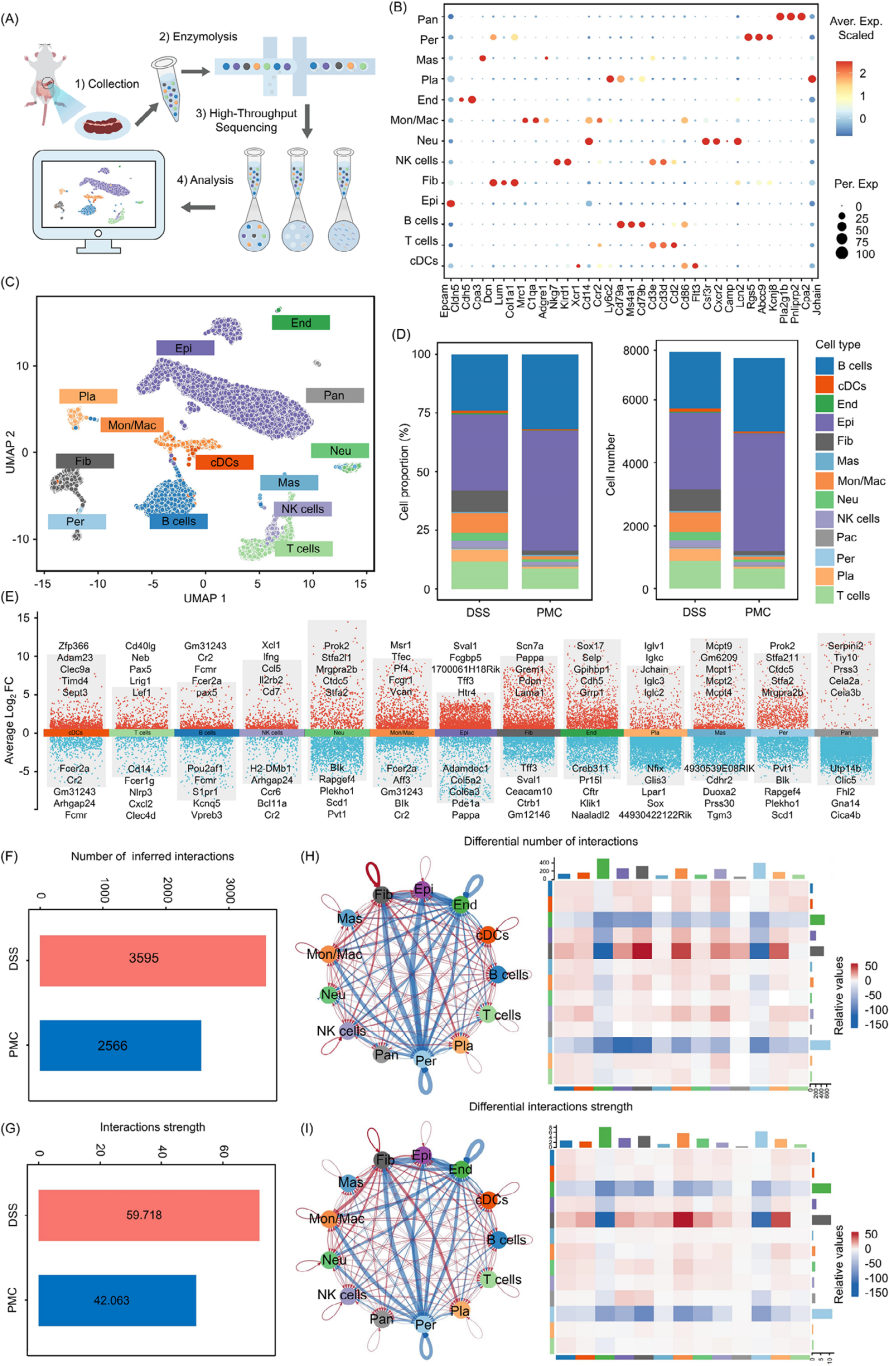
Single‐cell transcriptomic analysis of colonic tissues from DSS‐ and PMC‐treated mice after 13 days. A) Graphical overview of the scRNA‐seq experimental design. B) Dot plots displaying 13 cellular clusters with 35 signature gene expressions. The size of each dot represents the proportion of cells expressing a specific marker, and the color gradient indicates the mean expression levels of the markers (log1p transformed). C) Uniform Manifold Approximation and Projection (UMAP) plot showing the 13 identified main cell types in the colon of PMC‐treated mice, with each point representing an individual cell. D) Relative proportions and absolute numbers of each cellular cluster. E) Multi‐group volcano plot highlighting the top 5 most highly expressed genes and the top 5 least expressed genes across the 13 cellular clusters. F) Number of inferred ligand–receptor interactions and G) interaction strength of ligand–receptor pairs between the PMC‐treated and DSS‐treated groups. H) Differential ligand–receptor interactions (number) and I) differential interaction strength between the PMC‐treated and DSS‐treated groups, visualized in circle plots and heatmaps (red indicates increased interactions, blue indicates decreased interactions). In the circle plot, colored dots and squares represent different cellular clusters, with line thickness reflecting the number or strength of ligand–receptor pairs, and loops indicating autocrine circuits. In the heatmap, colored squares represent cellular clusters, with the color gradient reflecting the number or strength of ligand–receptor pairs. Abbreviations: cDCs, Conventional Dendritic Cells; Pla, Plasma Cells; Neu, Neutrophils; Mon, Monocytes; Mac, Macrophages; Epi, Epithelial Cells; Fib, Fibroblasts; End, Endothelial Cells; Mas, Mast Cells; Per, Pericytes; Pan, Pancreatic Alveolar Cells.

To impartially characterize the colonic landscape after PMC treatment for induced colitis, with a particular emphasis on PMC's regulatory effects on the colonic microenvironment during recovery, we conducted comprehensive single‐cell sequencing of intestinal tissues treated with and without PMC using a droplet‐based scRNA‐seq platform (10x Genomics) at day 13 (**Figure** **7**A). Seurat v5 was employed to analyze the sequencing results, and after excluding cells with excessively low RNA content or high mitochondrial gene expression, a total of 14872 cells were retained, comprising 7532 cells from the untreated DSS group and 7340 cells from the PMC‐treated group. Following dimensionality reduction via Uniform Manifold Approximation and Projection (umap), 13 subclusters were identified from the entire cell population, encompassing B cells (Cd79a, Cd79b), conventional dendritic cells (cDCs; Cd86), endothelial cells (Cldn5, Cdh5), epithelial cells (Epcam), fibroblasts (Col1a1, Dcn, Lum), mast cells (Cpa3), monocytes/macrophages (Mrc1, C1qa, Adgre1, Cd14, Ccr2, Ly6c2), neutrophils (Csf3r, Cxcr2, Camp, Lcn2), NK cells (Nkg7, Klrd1), pancreatic alveolar cells (Pla2g1b, Pnliprp2, Cpa2), pericytes (Rgs5, Adcc9, Kcnj8), plasma cells (Jchain), and T cells (Cd3e, Cd3d, Cd2) (Figure 7B,C). As anticipated, the numbers and proportions of immune cells (such as monocytes/macrophages, neutrophils, NK cells and T cells) dominated in the control group (DSS group) but significantly decreased after PMC treatment, while the numbers and proportions of epithelial cell notably increased in the PMC‐treated group, which indicated that PMC treatment may suppress DSS‐induced intestinal inflammation while promoting colonic epithelial cell regeneration (Figure 7D). In Figure 7E and Figure S17 (Supporting Information), multi‐group volcano and bubble plots showing the top 5 highly or poorly expressed genes and the top 5 significantly enriched pathways for these 13 cell types, respectively, further validating the accuracy of cell typing. In addition, we explored the cellular communication interaction and strength between 13 cell types under different treatments through cellchat v2, and constructed a ligand‐receptor, multimerization, and cofactor‐based cellular communication network. In Figure 7F,G, we found that cell communication (ligand–receptor pairs) interactions and strength were weaker in the PMC‐treated group compared to the control group. However, interestingly, cellular communication networks result in increased interactions and strength between epithelial cells and B cells, conventional dendritic cells, fibroblasts, mast cells, monocytes/macrophages, neutrophils, NK cells, pancreatic alveolar cells, pericytes, plasma cells and T cells, while interactions and strength with pericytes and endothelial cells decreased (Figure 7H,I). Although there was a decrease in the level of immune cell infiltration and an increase in the number of epithelial cells in the PMC group, there was an increase in the interaction and strength of communication between the immune cells and the epithelium. These results may imply that during reduced inflammation, intestinal epithelial cells had stronger communication with immune cells to coordinate tissue repair, restore barrier function, or promote immunomodulation, which was part of intestinal barrier repair.

**Figure 8:**
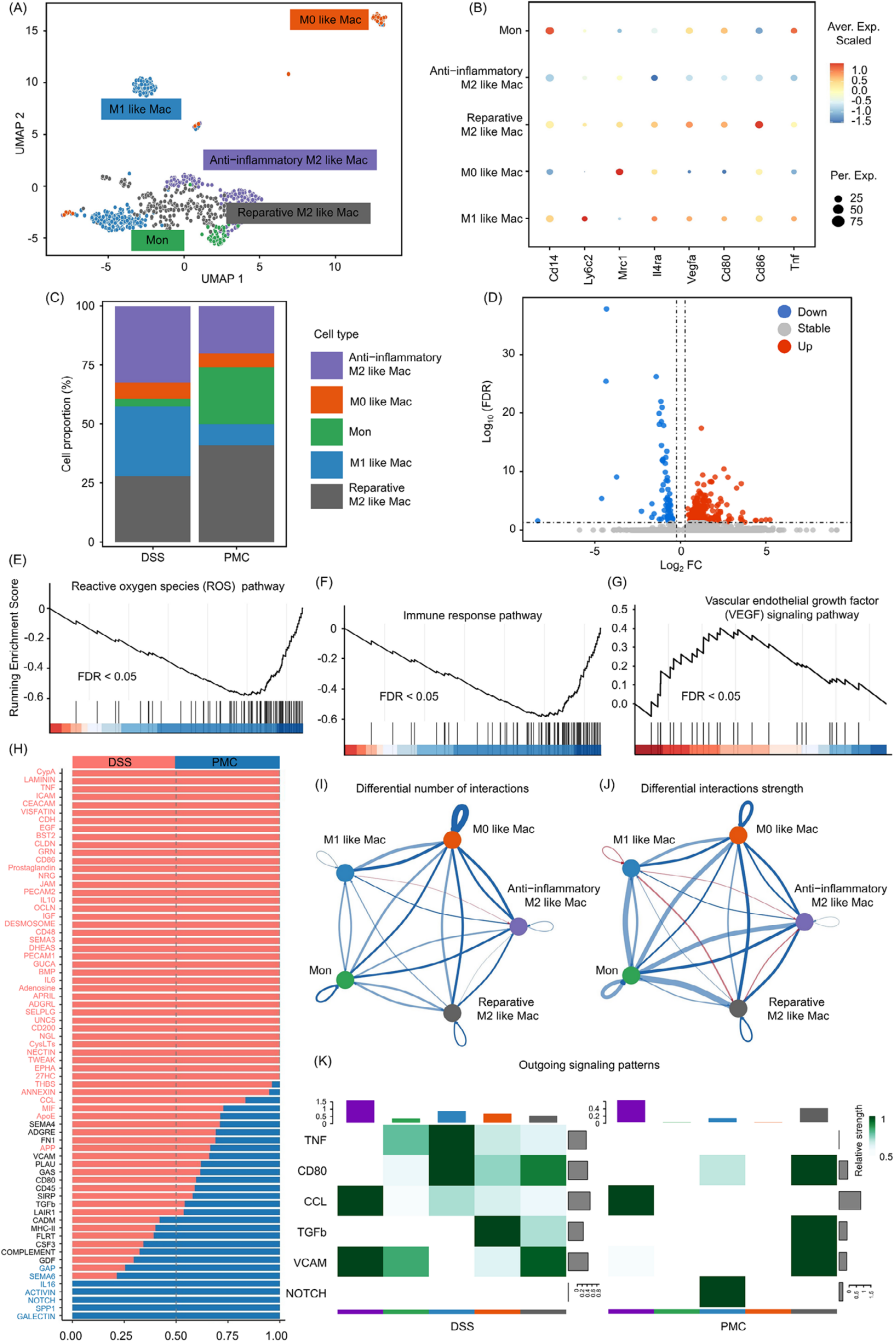
Distinct clusters of Mon/Mac cells in the colonic tissues of mice treated with DSS and PMC. A) UMAP analysis identifying 5 main subclusters of Mon/Mac cells. B) Dot plots showing marker gene expressions for the 5 cellular clusters, with eight signature genes. C) Relative proportions of each cellular cluster in the re‐clustering of Mon/Mac cells. D) Volcano plot highlighting DEGs in the PMC‐treated group compared to the DSS‐treated group. GSEA for E) ROS‐related genes, F) inflammation‐related genes, and G) repair‐related genes in the PMC‐treated group compared to the DSS‐treated group. H) Relative information flow of differentially active signaling pathways between the PMC‐ and DSS‐treated groups (two‐sided Wilcoxon test; FDR > 0.05). Genes marked in red indicate greater proportional significance in the DSS group, while genes marked in blue indicate greater significance in the PMC group. I) Network plot showing differential I) number and interactions J) strength of interactions in the 5 cellular clusters (red: up‐regulated in PMC‐treated group; blue: down‐regulated in PMC‐treated group). K) Heatmap illustrating outgoing signaling patterns in the PMC‐ and DSS‐treated groups.

Macrophages have a dual role in the different stages of inflammation. During acute inflammation, they promote inflammation, while after the inflammation subsides, they turn to support tissue repair.^[^ ^35^ ^]^ Therefore, we extracted the monocyte/macrophage subset from the total cell population and further classified them into monocytes (Cd14, Ly6c2), M0 macrophages, M1 macrophages (Cd80, Cd86, Tnf), anti‐inflammatory M2 macrophages (Mrc1), and reparative M2 macrophages (Il4ra, Vegfa) to further understand PMC's effects on macrophage function during the recovery phase of IBD (**Figure** **8**A,B). In the PMC‐treated group, both M1 and anti‐inflammatory M2 macrophage proportions declined, whereas reparative M2 macrophage proportions increased, suggesting that PMC treatment not only inhibits intestinal inflammation but also promotes the adoption of a reparative phenotype in M2 macrophages to facilitate injury recovery (Figure 8C). In addition, a total of 320 differentially expressed genes (DEGs) were obtained by differential analysis (PMC‐tread group/DSS‐treat group), of which 251 DEGs were up‐regulated and 69 DEGs were down‐regulated (Figure 8D). All Genes were analyzed by GSEA enrichment, which revealed a general downregulation of DEGs in the ROS pathway (Figure 8E) and immune response pathway (Figure 8F) and a general upregulation in the VEGF pathway (Figure 8G) in the PMC group. By identifying and visualizing conserved and specific signaling pathways in cellchat v2, we further found that the proportion of inflammation‐associated pathways (IL‐6, TNF, CCL) decreased and the proportion of repair signaling pathways (NOTCH) increased in the PMC group (Figure 8H). Meanwhile, we used cellchat v2 to find that the cell communication interactions and strength were weaker in the PMC‐treated group compared to the control group between the 5 re‐population of the cell types (Figure S18, Supporting Information), and there was a general decrease in the cell communication interactions and strength of re‐clustered Mon/Mac cells (Figures 8I,J). In Figure 8K, signal flow patterns of cells showed that TNF, CD86, CCL, and VCAM pathways in inflammation‐associated macrophage M1 signaled were weaker in the PMC‐treat group compared to the control group, and NOTCH pathway in inflammation‐associated macrophage M1 signaled more strongly compared to the DSS‐treat group. Moreover, TGF‐β and VCAM pathways in repair‐associated macrophage M2 signaled more strongly compared to the DSS‐treat group (Figure S19, Supporting Information). In the inflammatory response of IBD, the TNF‐α, CD86, and CCL pathways interact in multiple ways to drive disease progression: 1) TNF‐α is a core pro‐inflammatory factor in IBD that enhances immune responses and tissue damage through multiple signaling pathways and is an important target for anti‐inflammatory therapies.^[^ ^36^ ^]^ 2) CD86^+^ Macrophages, as resident mucosal macrophages, play a key role in driving chronic inflammation.^[^ ^37^ ^]^ 3) CCL chemokines, on the other hand, are mainly responsible for regulating immune cell migration and driving local inflammatory responses and immune cell infiltration.^[^ ^38^ ^]^ VCAM plays a key role in both inflammatory and reparative processes by promoting immune cell migration and adhesion to drive the inflammatory response during the inflammatory phase, and by supporting tissue recovery by directing reparative cells, promoting angiogenesis and tissue regeneration during the reparative phase.^[^ ^39^ ^]^ Additionally, as an anti‐inflammatory factor, TGF‐β helps to suppress excessive immune responses, maintain immune tolerance in the gut, and promote tissue repair.^[^ ^40^ ^]^ Based on the above results, we found that during the recovery phase of IBD, reparative M2 macrophages were overrepresented, and inflammatory M1 macrophages were underrepresented in the PMC group. Additionally, ROS and inflammation‐related pathways, as well as inflammatory factors, were inhibited, while reparative pathways and anti‐inflammatory factors were activated. These findings suggest that PMC plays a crucial role in remodeling macrophage function through multiple biological pathways, which is essential for intestinal barrier repair.

**Figure 9:**
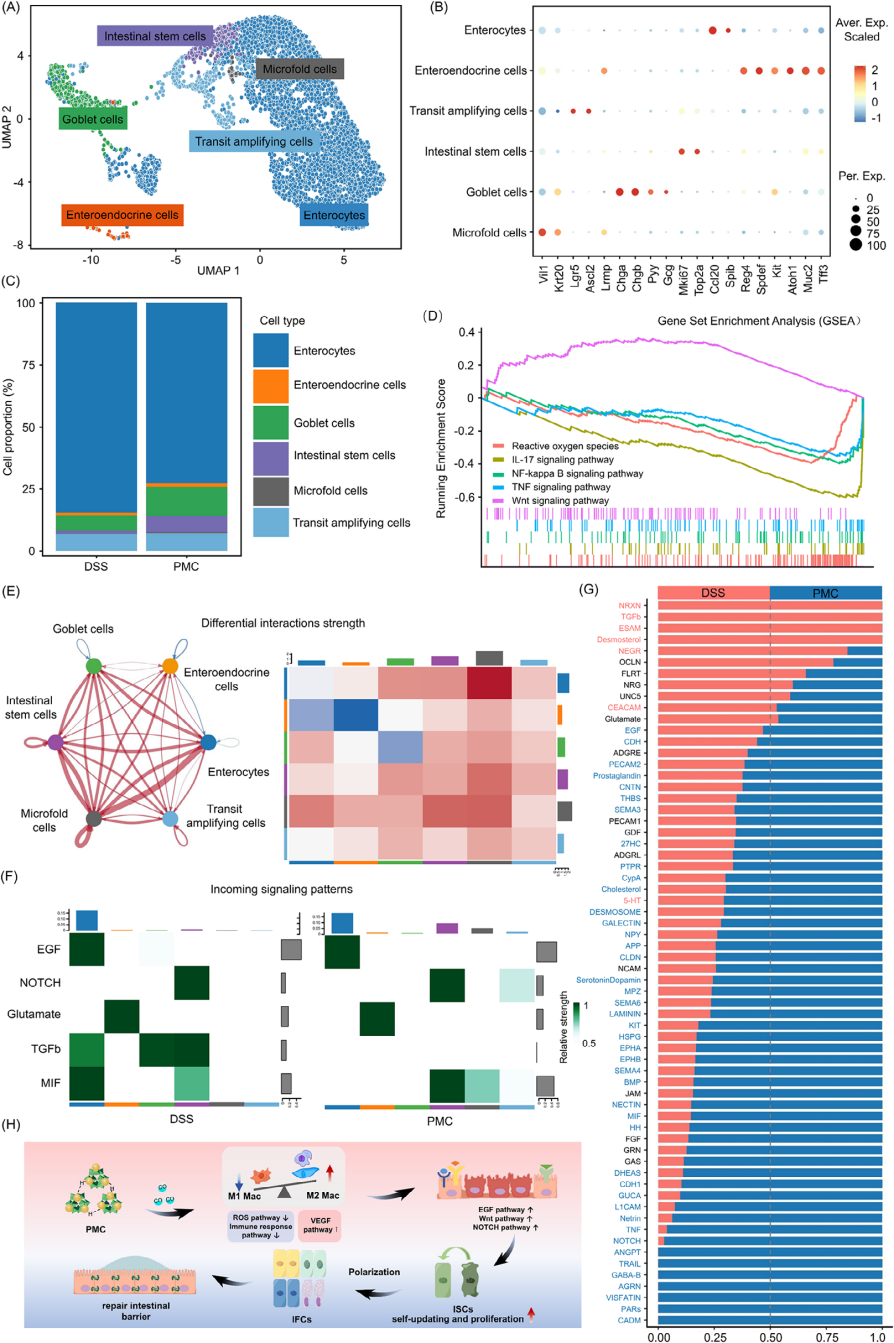
Distinct clusters of epithelial (Epi) cells in the colonic tissues of mice treated with DSS and PMC. A) UMAP analysis identifying 6 main subclusters of Epi cells. B) Dot plots showing marker gene expressions for the 6 cellular clusters, with nineteen signature genes. C) Relative proportions of each cellular cluster in the re‐clustered Epi cells. D) GSEA of Epi cells in the PMC‐treated group compared to the DSS‐treated group. E) the interaction strength of ligand–receptor pairs between the PMC‐treated and DSS‐treated groups. F) Heatmap showing incoming signaling patterns in the DSS‐treated group and PMC‐treated groups. G) Relative information flow of differentially active signaling pathways between the PMC‐ and DSS‐treated groups. H) Schematic illustration of the mechanisms underlying CO‐mediated intestinal barrier repair.

Given that epithelial cells are a major component of the colon with multiple functions such as barrier protection, absorption and secretion, immune defense, and tissue repair, especially in pathological conditions such as inflammatory bowel disease (IBD), where epithelial cell damage and repair are key factors in maintaining intestinal health, we further analyzed the composition of the epithelial cells. Among all 6182 epithelial cells, 6 epithelial subclusters were identified: enterocytes (Vil1, Krt20), enteroendocrine cells (Chga, Chgb, Pyy, Gcg), goblet cells (Atoh1, Muc2, Tff3), intestinal stem cells (Lgr5, Ascl2), Microfold cells (Ccl20, Spib), and transit‐amplifying cells (Mki67, Top2a) (**Figure** **9**A,B). In Figure 9C, Goblet cells secrete mucus to form a protective mucus layer, crucial for intestinal barrier protection and repair,^[^ ^41^ ^]^ and their proportion increased in the PMC‐treated group. Located at the crypt base, intestinal stem cells are responsible for colonic cell renewal. They produce new cells through mitosis, which gradually differentiate into transit‐amplifying cells, and further differentiate into various epithelial cells, including enterocytes and goblet cells.^[^ ^42^ ^]^ Elevated proportions of stem cells and transit‐amplifying cells after PMC treatment suggest that PMC treatment promotes intestinal mucosal barrier regeneration and repair by inducing epithelial cell differentiation. Furthermore, we explored the enrichment of all genes in the pathway by GSEA enrichment analysis and found that all genes in the ROS pathway, IL‐17 signaling pathway, NF‐κB signaling pathway and TNF signaling pathway were generally down‐regulated, while in WNT signaling pathway were up‐regulated (Figure 9D), which implied that oxidative damage and inflammation‐related signaling pathways were inhibited in the PMC‐treated group compared to the control group of epithelial cells and were in a state of proliferation and differentiation. By differential enrichment analysis, we obtained a total of 6173 DEGs, of which 5316 were up‐regulated and 857 were down‐regulated, and by KEGG enrichment analysis, we found that they were closely related to multiple pathways, including Inflammation‐related, oxidative stress‐related, and repair‐related pathways (Figure S20A,B, Supporting Information). Meanwhile, we utilized cellchat v2 to analyze cell‐cell communication in the DSS and PMC‐treated groups to uncover potential regulatory interactions between 6 epithelial subclusters in epithelial cells. Interestingly, unlike the initial clustering and monocyte macrophage re‐clustering, cell communication interactions and strength were stronger in the PMC‐treated group compared to the DSS‐induced group (Figure S20C, Supporting Information). Moreover, interactions and strength of cellular communication were generally elevated in PMC‐treat group compared DSS‐treat group (Figure 9E; Figure S21, Supporting Information), and signal flow patterns of cells revealed that in epithelial cells, which were the recipients of the signaling afferent pathway, the TGF‐β signaling pathway had a relative intensity of 0 in the PMC group for intestinal stem cells and enterocytes, and the NOTCH signaling pathway had a high relative intensity in both the PMC and DSS groups (Figure 9F). TGF‐β plays an important role in post‐inflammatory tissue repair, but if it is persistently expressed, it may lead to an excessive repair response, particularly by promoting fibroblast proliferation and collagen deposition, which may lead to intestinal fibrosis.^[^ ^43^ ^]^ Notch signaling plays an important role in the proliferation and differentiation of intestinal stem cells.^[^ ^44^ ^]^ In IBD, the function of intestinal stem cells may be inhibited due to inflammatory injury, and the regulation of Notch and EGF signaling contributes to the recovery and regeneration of stem cells and promotes the repair of the intestinal epithelium.^[^ ^42^ ^]^ In Figure 9G, relative information flow result showed that TGF‐β was highly expressed in the DSS group, while NOTCH and EGF were highly expressed in the PMC group. Based on the results of the analysis of intestinal epithelial cell repopulation, in the PMC group, epithelial cells had the highest number in the PMC group and the proportion of intestinal stem cells, goblet cells, and resorption was increased, while the NOTCH signaling pathway was highly activated while the TGF‐β signaling pathway was inhibited, which facilitated the proliferation and differentiation of intestinal stem cells as well as inhibited the generation of fibrosis due to excessive repair.

In summary, as shown in Figure 9H, we conclude that CO promotes intestinal repair in IBD through the following mechanisms: PMC first releases CO, which suppresses ROS signaling and immune response pathways while activating the VEGF pathway. This cascade of events fosters an anti‐inflammatory and reparative microenvironment, characterized by the downregulation of M1‐type macrophages (pro‐inflammatory) and an increase in M2‐type macrophages (anti‐inflammatory and reparative). Subsequently, CO activates downstream signaling pathways, including EGF, WNT, and NOTCH, to enhance the self‐renewal and functional recovery of intestinal stem cells (ISCs). Furthermore, CO regulates the polarization of ISCs, ensuring their proper differentiation and the development of absorptive cells, goblet cells, and Paneth cells. Through this coordinated regulatory network, CO helps preserve the integrity and functionality of the intestinal barrier, ultimately restoring intestinal homeostasis.

## 3 Conclusion

This study introduces a novel CO‐releasing POMs nanozyme (PMC), synthesized by coordinating MnBr(CO)₅ with molybdenum‐based POMs nanoclusters. PMC exhibits several superior properties for the treatment of IBD, including targeted accumulation at IBD sites, efficient ROS scavenging, and CO release in response to ROS. These characteristics contribute to its antioxidant, anti‐inflammatory, microbiota‐modulating, and intestinal barrier‐repairing effects. Both in vitro and in vivo experiments demonstrated that PMC effectively mitigates oxidative stress, reduces inflammation, and promotes epithelial barrier repair, indicating significant therapeutic efficacy in ulcerative colitis treatment. 16S rRNA sequencing revealed that PMC remodeled the gut microbiota, while scRNA‐seq validated that PMC significantly enhanced the immune balance and tissue repair in the colonic tissues of DSS‐induced colitis mice. This was achieved by decreasing the proportion of pro‐inflammatory M1 macrophages, inhibiting ROS‐ and immune‐related inflammatory signaling pathways, and significantly increasing the proportion of reparative M2 macrophages and intestinal stem cells. Additionally, PMC activated the VEGF signaling pathway in macrophages and the NOTCH signaling pathway in intestinal stem cells. Overall, PMC effectively restored intestinal barrier integrity by modulating gut mucosal immunity and microbial homeostasis, highlighting its potential as a therapeutic agent for IBD. This study proposes PMC as a promising multifunctional therapeutic strategy that offers novel insights and approaches to enhance the clinical management of IBD.

## Conflict of Interest

The authors declare no conflict of interest.

## Supporting information



Supporting Information

## Data Availability

The data that support the findings of this study are available from the corresponding author upon reasonable request.
