# Correction to “CO‐Releasing Polyoxometalates Nanozyme With Gut Mucosal Immunity and Microbiota Homeostasis Remodeling Effects for Restoring Intestinal Barrier Integrity”

**DOI:** 10.1002/advs.75606

**Published:** 2026-05-20

Hongyang Lu, Qiang Zhou, Jiayu Li, Shengming Xu, Li Yu, Yinci Zhu, He Zhang, Chengge Shi, Tianci Zuo, Mengzhu Xu, Mingli Su, Yanmei Zhang, Rongdang Hu, Quazi T. H. Shubhra^*^, Hui Deng^*^, Xiaowen Hu^*^, Xiaojun Cai* “CO‐Releasing Polyoxometalates Nanozyme with Gut Mucosal Immunity and Microbiota Homeostasis Remodeling Effects for Restoring Intestinal Barrier Integrity,” *Advanced Science* 12, no. 17 (2025): e2500116. https://doi.org/10.1002/advs.202500116.

Following our recent thorough self‐review after publication, we have identified one unintentional error in the published manuscript. In Figure 8, two panels were mistakenly labeled with E, and one panel was mistakenly labeled with F; correspondingly, two images corresponding to these three panels were misused. These errors occurred during the final stage of figure preparation and, regrettably, were overlooked before submission.


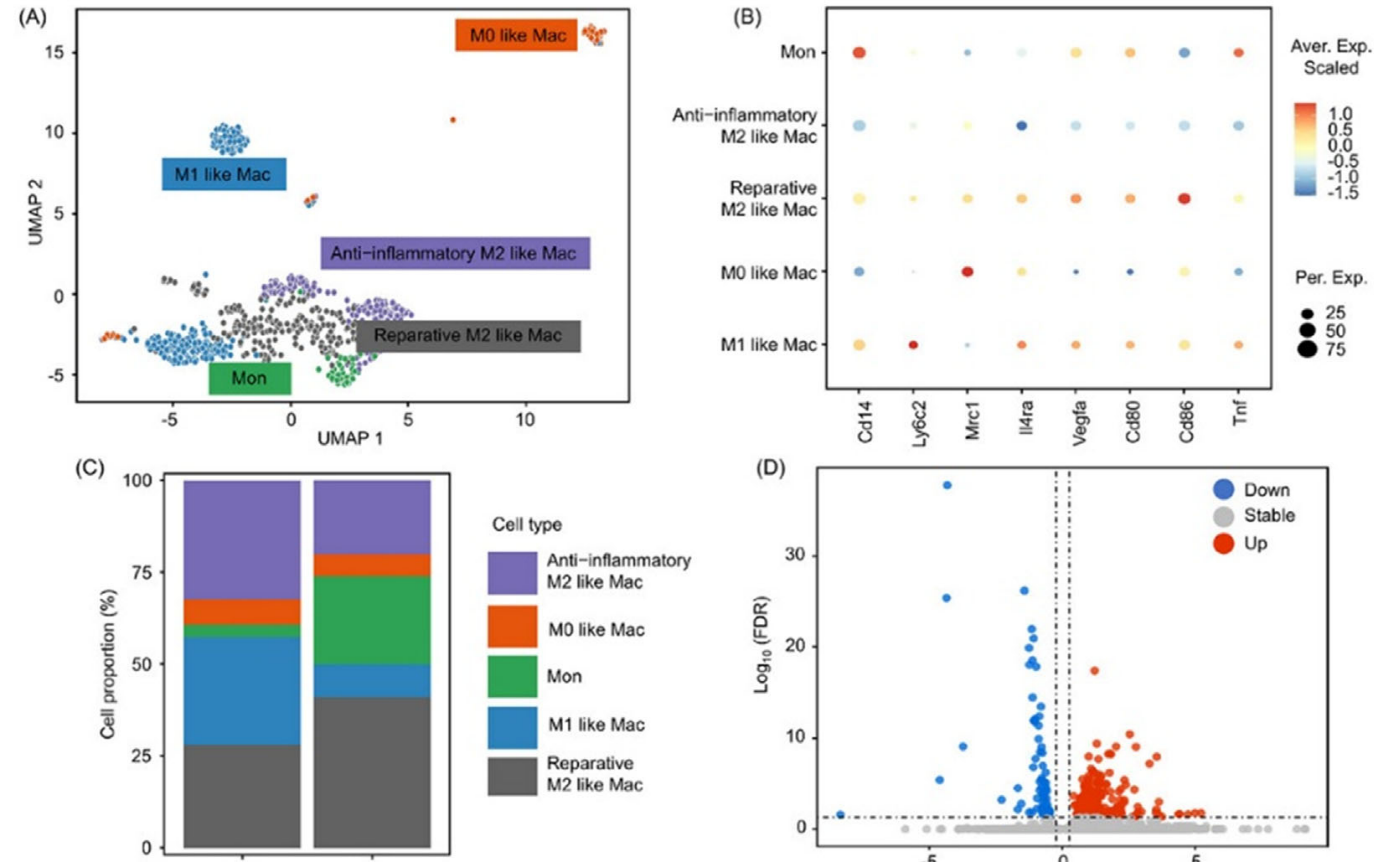



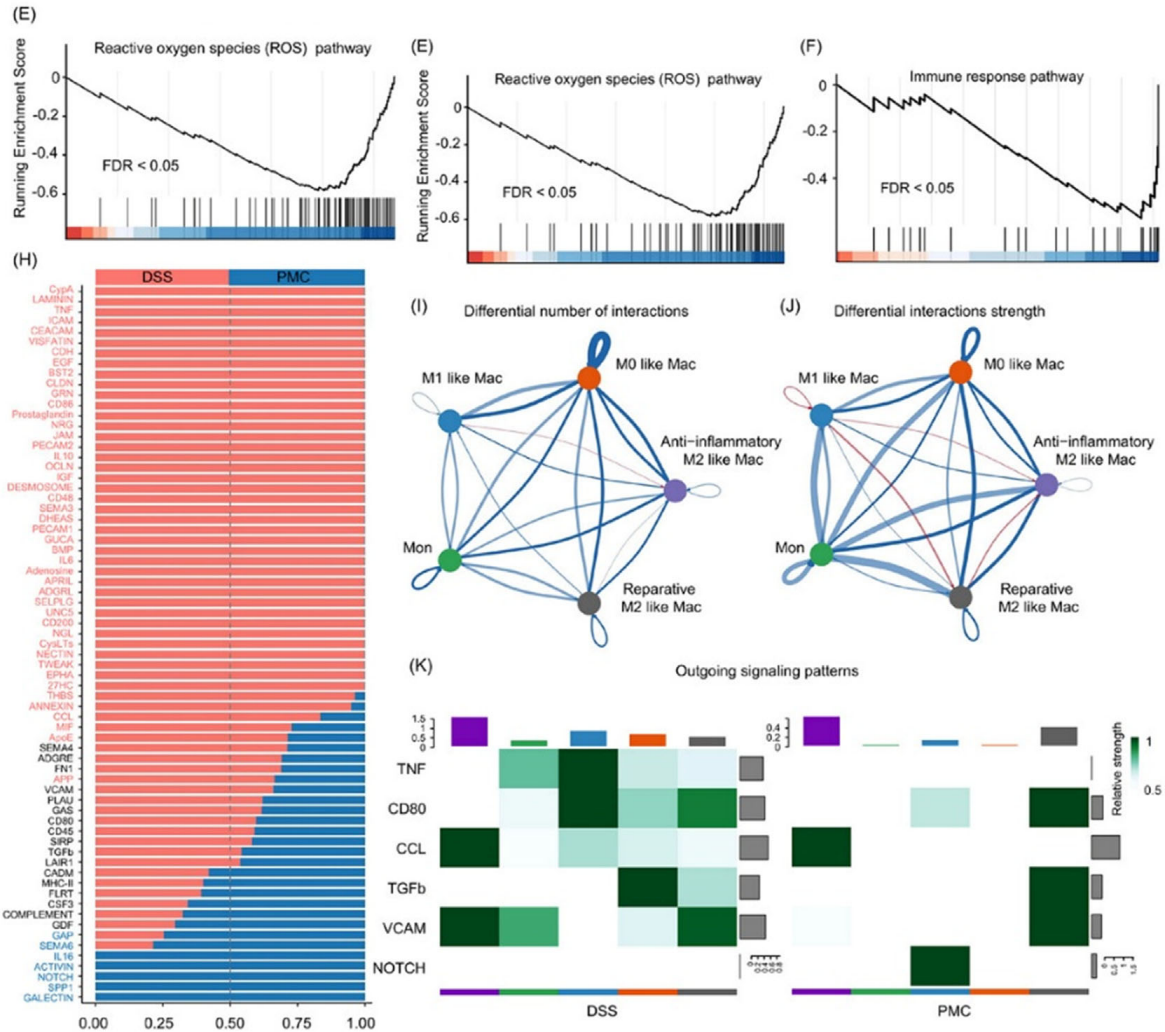


**InFigure 8**. E) ROS‐related genes, F) inflammation‐related genes.

These are the correct images for Figure 8E, 8F, and 8G that should replace the published Figure 8E, 8E, and 8F.


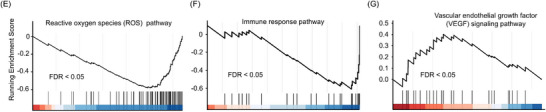


These corrections do not affect the overall findings and conclusions of the paper.

We sincerely apologize for these errors.

