# Supplementary material for: CO‐Releasing Polyoxometalates Nanozyme with Gut Mucosal Immunity and Microbiota Homeostasis Remodeling Effects for Restoring Intestinal Barrier Integrity
Source: Adv Sci (Weinh). 2025 Mar 13;12(17):2500116. doi: 10.1002/advs.202500116 (PMC12061238; doi:10.1002/advs.202500116)
Supplement: Supplementary file 1 — Supporting Information [file ADVS-12-2500116-s001.pdf]

ADVANCED SCIENCE
Open Access

Supporting Information

for *Adv. Sci.*, DOI 10.1002/advs.202500116

CO-Releasing Polyoxometalates Nanozyme with Gut Mucosal Immunity and Microbiota Homeostasis Remodeling Effects for Restoring Intestinal Barrier Integrity

Hongyang Lu, Qiang Zhou, Jiayu Li, Shengming Xu, Li Yu, Yinci Zhu, He Zhang, Chengge Shi, Tianci Zuo, Mengzhu Xu, Mingli Su, Yanmei Zhang, Rongdang Hu, Quazi T. H. Shubhra, Hui Deng*, Xiaowen Hu* and Xiaojun Cai**

Supporting Information

CO-Releasing Polyoxometalates Nanozyme with Gut Mucosal Immunity and Microbiota Homeostasis Remodeling Effects for Restoring Intestinal Barrier Integrity

Hongyang Lu, Qiang Zhou, Jiayu Li, Shengming Xu, Li Yu, Yinci Zhu, He Zhang, Chengge Shi, Tianci Zuo, Mengzhu Xu, Mingli Su, Yanmei Zhang, Rongdang Hu, Quazi T. H. Shubhra, Hui Deng*, Xiaowen Hu*, Xiaojun Cai**

H. Lu, J. Li, S. Xu, L. Yu, Y. Zhu, H. Zhang, C. Shi, T. Zuo, M. Xu, M. Su, Y. Zhang, R. Hu, H. Deng, X. Hu, X. Cai
School and Hospital of Stomatology, Wenzhou Medical University, Wenzhou 325027, China
E-mail: huideng@wmu.edu.cn; huxiaowen@wmu.edu.cn; cxj520118@wmu.edu.cn; cxj520118@njtech.edu.cn

Q. Zhou
Ruian People's Hospital, The Third Affiliated Hospital of Wenzhou Medical University, Wenzhou 325016, China

Q.T.H. Shubhra
Institute of Chemistry, University of Silesia in Katowice, Szkolna 9, Katowice, Poland
Email: tanminul-haque-shubra.quazi@us.edu.pl

Experimental section

Materials:

Ammonium molybdate, sodium dihydrogen phosphate (NaH_2PO_4), ascorbic acid, dexamethasone, and $MnBr(CO)_5$ were purchased from Shanghai Aladdin Biochemical Technology Co., Ltd. The ROS DCFH-DA probe was obtained from Solarbio Bioscience. DSS salt and LPS were sourced from Sigma-Aldrich (USA). Cell-Light EdU Apollo 567 imaging kit was obtained from Guangzhou Ribobio. Calcein/PI cell viability and cytotoxicity assay kits were obtained from Beyotime Biotech (China). Detection kits for CAT, SOD, GSH-Px,·OH, and ABTS were provided by Grace Biotech Co., Ltd. Hoechst 33342 was sourced from Keygen Biotech Co., Ltd. (Nanjing, China). The mouse HO-1 ELISA kit was sourced from Cusabio (China). Antibodies targeting HO-1, 8-OHDG and PE-labeled anti-mouse F4/80, along with ELISA kits for mouse TNF-α, IL-1β, IL-6, IL-10, and MPO were purchased from Proteintech (USA). Annexin V-FITC/PI apoptosis kit and L-012 sodium salt were purchased from APEBio (USA). The mouse HbCO ELISA kit was obtained from Meimian Biotech (Beijing, China). FITC-labeled anti-mouse CD86 antibody was obtained from Elabscience (China), and FITC-labeled anti-mouse CD206 antibody was purchased from BioLegend. RAW264.7 and Caco-2 cells were provided by Wuhan Procell Life Science and Technology Co.

Synthesis and characterization of PMCs

First, a solution of 5 mM ammonium molybdate and 2.92 mM NaH_2PO_4 was prepared by dissolving the compounds in 20 mL of deionized water and stirring vigorously for 20 minutes. Subsequently, 400 mg of ascorbic acid was introduced into the mixture, and the reaction was allowed to proceed for 90 minutes. After the reaction was completed, 80 mL of anhydrous ethanol was added to precipitate the product. The mixture was then centrifuged at 10,000 rpm for 15 minutes, and the supernatant was poured off. The sediment was washed three times with a solution of anhydrous ethanol and water, followed by freeze-drying. The dried powder was stored for further use. The structure of the synthesized polyoxometalates (POMs) was characterized using fourier transform infrared (FTIR) spectroscopy, X-ray photoelectron spectroscopy (XPS), and UV-visible absorption spectroscopy. The molybdenum (Mo) content in POMs was measured and calculated through inductively coupled plasma mass spectrometry (ICP-MS).

Next, 60 mg or 100 mg of $MnBr(CO)_5$ and 300 mg of POMs were dispersed in methanol and stirred in the dark at 25℃ for 12 hours. The methanol was subsequently evaporated with a

rotary evaporator, and the residue was resuspended in distilled water. The suspension was freeze-dried to obtain a green flocculent product, which was designated as PMC. Dynamic light scattering (DLS, NanoTM 90) was employed to measure the particle size and zeta potential of PMC. The surface morphology and distribution of CORM-401 on PMC were observed using transmission electron microscopy (TEM, JEM-2100 Plus) and energy-dispersive X-ray spectroscopy (EDS). The manganese (Mn) content in PMC was measured through ICP-MS to calculate the loading efficiency and loading capacity of $MnBr(CO)_5$.

We subsequently assessed the ROS scavenging capacity of PMC in solution. First, the catalase-like (CAT) activity of PMC was evaluated using a catalase assay kit. Specifically, 10 μL of PMC at different concentrations (Mo = 50 or 100 μg/mL) was reacted with a solution containing approximately 100 μM H_2O_2 for 10 minutes. Absorbance was then measured at 510 nm to calculate CAT activity. The superoxide dismutase (SOD)-like activity was determined by the WST-8 method: 20 μL of PMC was allowed to react with superoxide anions ($O_2^{-}\cdot$) generated by xanthine oxidase (XO) for 30 minutes, followed by measuring absorbance at 450 nm. Finally, the ·OH scavenging rate was measured by reacting hydroxyl radicals (·OH) generated through the Fenton reaction with 50 μL of PMC for 25 minutes. After color development with salicylic acid, absorbance was measured at 510 nm to calculate PMC's ·OH scavenging capacity.

The CO release behavior of PMC was evaluated following the method reported by Urara Hasegawa. First, a sample vial containing 2 mL of PMC solution was placed alongside a CO gas detector inside a glass container with a sealable lid. The top of the container was sealed with a rubber stopper, and all interfaces were further secured using sealing film and vacuum grease to ensure the entire detection system was airtight. Subsequently, different concentrations of H_2O_2 were injected into the PMC solution using a syringe, and the concentration of CO gas was recorded at various time points. The amount of CO released was calculated according to the formula reported in the literature.[1]

Cell viability of PMC

In this study, RAW264.7 cells and Caco-2 cells were maintained using macrophage-specific and Caco-2-specific media provided by Wuhan Pricella Biotech. "Normal macrophages" are defined as RAW264.7 cells not treated with LPS, whereas "activated macrophages" refer to RAW264.7 cells exposed to LPS (5 μg/mL) for 24 hours.

The cytotoxicity of PMC on RAW264.7 and Caco-2 cells were evaluated using the CCK-8 assay and live/dead cell staining. Specifically, RAW264.7 cells (1×10^4 cells/well) and Caco-

2 cells (1×10^4 cells/well) were plated in 96-well plates and incubated overnight.. The medium was then substituted with PMC-containing medium (at concentrations ranging from 10 to 640 μg/mL), followed by incubation at 37°C for 24 hours. After incubation, the supernatant was removed, and the cells underwent three washes with PBS. The CCK-8 reagent was then introduced, followed by measurement of absorbance at 450 nm using a microplate reader. Live and dead cells were also observed under an inverted fluorescence microscope (AxioObserver3, ZEISS) after staining with Calcein-AM and PI. Cells treated with PBS and POMs were used as controls.

The biocompatibility of PMC was further evaluated through a standardized hemolysis assay. Whole blood was obtained from healthy Sprague-Dawley rats via the retro-orbital method, and red blood cells (RBCs) were collected by centrifugation (3000 rpm, 15 min). A 500 μL aliquot of dd H_2O, PBS, POMs, or PMC was mixed with 500 μL of H_2O_2 (200 μM) and 20 μL of RBCs. The mixture was kept at 37°C for 4 hours., followed by centrifugation (3000 rpm, 15 min). The supernatant was gathered, and absorbance was measured at 542 nm with a microplate reader. The hemolysis rate (%) was calculated following a previously reported procedure. PBS was designated as the negative control, and dd H_2O as the positive control. POMs and PMC in the absence of H_2O_2 acted as material controls.

Intracellular CO imaging

The intracellular CO release was assessed based on the methods outlined by Michel et al. Briefly, activated RAW264.7 cells (3×10^5 cells/well) were plated in 35 mm glass-bottom dishes and incubated for 24 hours. The medium was subsequently swapped with serum-free DMEM for 1.5 hours to induce starvation. Subsequently, the cells were exposed to DMEM containing PMC at a concentration of Mo = 100 μg/mL for 4 hours. Following incubation, the cells were stained in sequence with 5 μM $PdCl_2$ and 5 μM FL-CO-1 (CO probe system) along with Hoechst 33342. CO imaging was observed using an inverted fluorescence microscope (AxioObserver3, ZEISS). The FL-CO-1 probe was excited at 490 nm, with emission measured at 512 nm. In parallel, the DCFH-DA probe was used to measure ROS levels in activated macrophages, with excitation and emission wavelengths set to 644 nm and 665 nm, respectively.

In vitro antioxidant of PMC

To assess the antioxidant effects of PMC, activated macrophages (2×10^5 cells/well) were cultured in a 6-well plate for 24 hours. After incubation, The cells underwent treatment with DMEM supplemented with PMC at a concentration of Mo = 100 μg/mL , followed by a further

24-hour incubation. The cells were then harvested and subjected to sonication on ice (300 W, 5 s on/25 s off cycles) for 30 minutes. After centrifugation, The supernatant was obtained, and the protein concentration was quantified using a BCA protein assay kit. The absorbance of the samples was recorded at 510 nm, 450 nm, and 412 nm, and the activities of CAT, SOD, and GSH-Px, along with the hydroxyl radical scavenging rate, were calculated following the manufacturer's protocols. In parallel, immunofluorescence staining was performed on PMC-treated activated macrophages using anti-8-OHdG and anti-HO-1 antibodies, and the cells were imaged using a laser confocal microscope. For the Western blot analysis of HO-1, cells were treated with RIPA buffer with protease inhibitors for protein extraction. Proteins were separated by electrophoresis, transferred onto membranes, blocked, and incubated with primary antibodies specific for HO-1. Protein bands were visualized using an enhanced chemiluminescence kit, and images were obtained with a gel documentation system. ImageJ software was used to quantify band intensity, with β-actin serving as the loading control. Cells treated with PBS, Dex-p, or POMs served as controls in all experiments. The specific primer sequences employed in this study are detailed in the supporting information (Table 3).

In vitro anti-inflammatory effect of PMC

The anti-inflammatory effects of PMC were assessed by treating activated macrophages as outlined above. The cells were then exposed to DMEM containing PMC for an additional 24 hours. Morphological changes were then observed under an inverted fluorescence microscope. Parallel cell samples were collected for quantitative analysis of TNF-α, IL-1β, IL-6, iNOS, Arg-1, and CD206 expression via real-time quantitative PCR (RT-qPCR). mRNA was purified from the lysed cell samples for RT-qPCR analysis., followed by reverse transcription, amplification, and quantification using a real-time PCR system. For flow cytometry, macrophages were washed, fixed, and incubated with anti-CD86 or anti-CD206 antibodies, followed by analysis on a Beckman flow cytometer (CytoFLEX). Cells treated with PBS, Dex-p, or POMs served as controls in all experiments. The primer sequences employed in this study are provided in the Supplementary Information (Table 3).

Effect of PMC on Promoting Intestinal Mucosal Barrier Repair

To assess the protective effect of PMC on Caco-2 cells in vitro, an oxidative damage model was established by inducing damage in Caco-2 cells using H_2O_2. Caco-2 cells (2×10^4 cells/well) were seeded in 96-well plates and cultured for 24 hours. Afterward, the cells were exposed to DMEM containing 1 mM H_2O_2 for 2 hours, followed by the addition of PMC at a

specific concentration. The cells were subsequently maintained for an additional 24 hours. Following treatment, supernatants were collected for lactate dehydrogenase (LDH) activity analysis using an LDH assay kit. In parallel, cell viability was evaluated by adding CCK-8 working solution, followed by absorbance measurement at 450 nm using a microplate reader. Cell viability and death were also observed using an inverted fluorescence microscope (AxioObserver3, ZEISS) after staining with Calcein-AM and propidium iodide (PI). Additionally, apoptosis in PMC-treated Caco-2 cells was evaluated using the Annexin V-FITC/PI Apoptosis Kit. Treated cells were harvested, washed with pre-cooled PBS, and centrifuged. The cell pellet was mixed with 500 μL of 1X Binding Buffer, to which 2 μL of Annexin V-FITC and 2 μL of PI were added. After dark incubation for 10 minutes, the samples were analyzed by flow cytometry.

To assess the proliferative effect of PMC on Caco-2 cells, the cells were treated according to the procedure outlined above. They were then incubated in fresh DMEM medium containing 10 mM EdU for 6 hours. Following incubation, the cells were fixed with 4% paraformaldehyde (PFA) and permeabilized with 0.3% Triton X-100, before being stained with Hoechst 33342 (5 μg/mL). EdU incorporation was analyzed under an inverted fluorescence microscope.

To evalute the wound-healing effect of PMC on Caco-2 cells, 5×10^4 cells/well were uniformly seeded into three-well Ibidi culture inserts and incubated at 37°C for 24 hours. After cell adhesion, a scratch of 0.5 mm width was made. The cells were then treated and cultured for an additional 24 hours following the steps mentioned above. The wound-healing process was observed using an inverted microscope at 0, 12, and 24 hours.

To evaluate the effect of PMC on tight junction protein expression in Caco-2 cells, the cells were cultured according to the steps described earlier. Total RNA was extracted, and the expression levels of ZO-1, Occludin, and Claudin-1 were quantitatively analyzed using RT-qPCR. The primer sequences employed in this study are provided in the supplementary information (Table 3).

For these experiments, the following groups were used as controls: 1) Caco-2 cells incubated in DMEM under normal conditions; 2) Caco-2 cells maintained in DMEM with 1 mM H_2O_2; 3) Caco-2 cells cultured in DMEM treated with Dex-p (2.5 μg/mL) or POMs (Mo=100 μg/mL).

Evaluation of the Therapeutic Effect of PMC on DSS-Induced Colitis in Mice

The animal experiments in this study received approval and supervised from the Animal Ethics Committee of Wenzhou Medical University (approval number: wydw 2024-0337).

Female BALB/c mice (6-8 weeks old) were purchased from Beijing SPF Biotech Co. and acclimatized for one week before the study. The mice received 3% (w/v) DSS in their drinking water for 5 days.[2] Following this, 24 mice were assigned to four groups (n = 6) and were given 3% (w/v) DSS throughout the treatment period.. On days 5, 7, 9, and 11, each mouse with colitis was administered a single rectal administration of 200 μL of either PBS, Dex-p (75 μg/mL), POMs (1.5 mg/mL), or PMC (1.5 mg/mL). Six normally reared healthy mice were used as controls. The mice were observed daily for changes in body weight and fecal conditions, and Disease Activity Index (DAI) scores were assigned according to previously established methods.

On day 11, the ROS scavenging capacity of PMC was evaluated by intraperitoneally injecting 80 μL of L-012 probe solution (6 μg/mL), followed by capturing luminescent images of the inflamed intestines using an in vivo imaging system (IVIS， Lumina XRMS Series III， PerkinElmer).[3] At the conclusion of the experiment, All mice were euthanized, and their colonic tissues were harvested to assess colonic length. Histological analysis was performed by washing, fixing, embedding, sectioning, and applying H&E staining to the colonic segments and essential organs. Histological scoring was carried out as per established guidelines.

To assess the antioxidant potential of PMC in vivo, colonic segments were homogenized and centrifuged, and CAT and SOD expression levels were measured using detection kits. The anti-inflammatory effects of PMC were evaluated by measuring the levels of IL-1β, TNF-α, IL-6, and MPO in the supernatant using ELISA kits. For immunofluorescence staining, colonic sections were labeled with anti-CD86 and CD206 antibodies and observed using a laser confocal microscope. To investigate the impact of PMC on the repair of the intestinal barrier in mice, colonic sections were immunofluorescence stained with anti-ZO-1, Occludin, and Claudin-1 antibodies, also observed with a laser confocal microscope. At the end of the experiment, blood samples were drawn from the orbital sinus of the mice to perform a hematological analysis and evaluate the biosafety of the entire treatment. Additionally, plasma samples were obtained, and HbCO levels were measured using a mouse HbCO ELISA kit.

Single-cell RNA sequencing (scRNA-seq)

10x Genomics Cell Ranger 7.1.0 is used to align and quantify raw reads within each cell with mm10 as the reference gene set. A total of 8,928 cells were identified in the DSS group, with 39,401 raw reads detected in each cell and a median gene count of 1,919. In the PMC group, 9,241 cells were identified with 37,456 raw reads detected per cell and a median gene count of 2,394. The data were then merged, preprocessed, and quality-controlled using Seurat

v5.[4] 14872 Cells (7,532 cells in the DSS group and 7,340 cells in the PMC group) with gene counts ranging from 250 to 7000, RNA counts exceeding 500, and the percentages of mitochondrial gene expression less than 15% were retained. The merged data were normalized and the top 2000 variable genes are identified, which exhibited substantial variability across cells and should be used for dimensionality reduction and clustering. The data were then embedded via uniform manifold approximation and projection (UMAP). We used FindNeighbors and FindClusters to construct the SNN graph, which was then used to identify subclusters. After determining the identity of each cell cluster, FindAllMarkers was employed to identify marker genes of each cluster. CellChat v2[5] was utilized to recognize overexpressed genes, compute cell communication probabilities, and then construct a cell communication network based on interactions involving ligands, receptors, polymers, and cofactors.

Microbiome Analysis

To assess the regulatory effects of PMC on gut microbiota, we conducted a study using 16S rRNA gene sequencing. At the end of the experiment, fresh fecal samples were collected from the colon segments of healthy mice, colitis mice without treatment, and colitis mice treated with PMC. The samples were rapidly frozen using dry ice and then placed in a -80°C ultra-low temperature freezer for storage. Total DNA was extracted from the samples using the E.Z.N.A. Soil DNA Kit. The quality and concentration of the extracted DNA were carefully evaluated using 1.0% agarose gel electrophoresis and a Nanodrop 2000 spectrophotometer. The V3-V4 region of the bacterial 16S rRNA gene was then amplified using specific primers on an ABI GeneAmp 9700 PCR thermocycler.[3] Following the standard protocol provided by Majorbio Bio-Pharm Technology Co., Ltd., the microbial composition was analyzed using the MiSeq Illumina sequencing platform.[2] Operational taxonomic units (OTUs) were assigned according to a 97% similarity cutoff., and sequences were clustered using UPARSE software (version 7.1). Representative sequences of each OTU were classified with reference to the Silva database, with a confidence threshold set at 0.7. Finally, all experimental data were analyzed using the Majorbio Biocloud platform.

Statistical Analysis

Results are expressed as the mean ± standard deviation (SD). All data represent at least three independent experiments. Statistical analysis was conducted using unpaired t-tests and one-way analysis of variance (ANOVA) as applicable. Data were analyzed using Prism software. A p-

value of less than 0.05 was considered statistically significant ($*p < 0.05$), with additional significance levels indicated as $**p \leq 0.01$ and $***p \leq 0.001$.

References

[1] G. Yang, M. Fan, J. Zhu, C. Ling, L. Wu, X. Zhang, M. Zhang, J. Li, Q. Yao, Z. Gu, X. Cai, *Biomaterials* **2020**, 255, 120155.

[2] Y. Zeng, M. Fan, Q. Zhou, D. Chen, T. Jin, Z. Mu, L. Li, J. Chen, D. Qiu, Y. Zhang, Y. Pan, X. Shen, X. Cai, *Adv. Funct. Mater.* **2023**, 33, 2304381.

[3] T. Jin, H. Lu, Q. Zhou, D. Chen, Y. Zeng, J. Shi, Y. Zhang, X. Wang, X. Shen, X. Cai, *Adv. Sci.* **2024**, 11, 2308092.

[4] Y. Hao, T. Stuart, M. H. Kowalski, S. Choudhary, P. Hoffman, A. Hartman, A. Srivastava, G. Molla, S. Madad, C. Fernandez-Granda, R. Satija, *Nat. Biotechnol.* **2024**, 42, 293.

[5] S. Jin, C. F. Guerrero-Juarez, L. Zhang, I. Chang, R. Ramos, C. H. Kuan, P. Myung, M. V. Plikus, Q. Nie, *Nat. Commun.* **2021**, 12, 1088.

Supplementary Figures and Tables

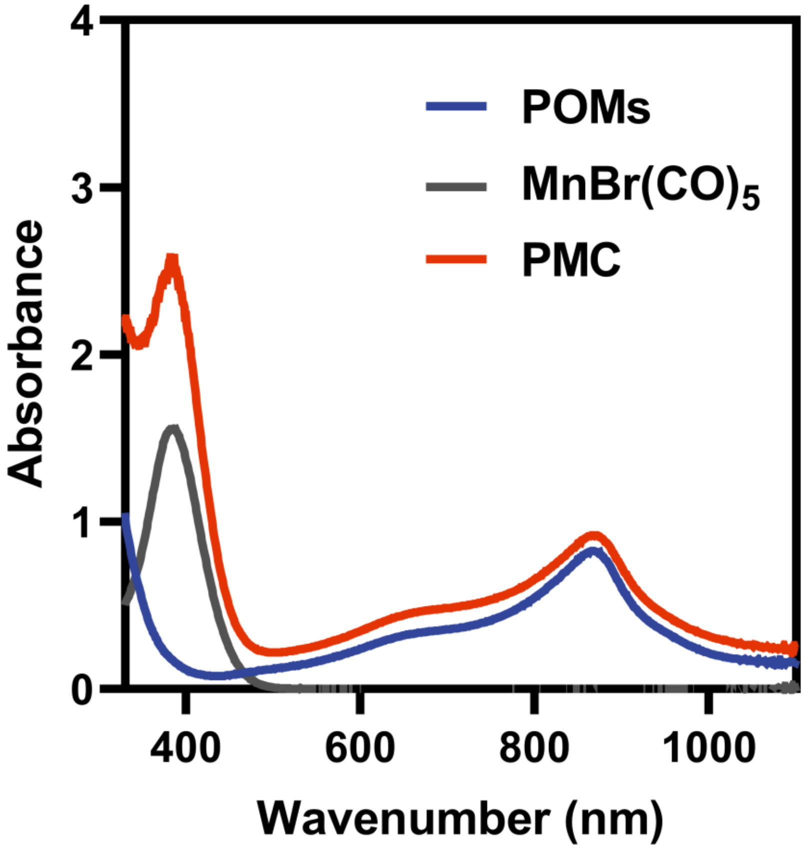

Figure S1. UV-vis spectra of POMs, $MnBr(CO)_5$ and PMC.

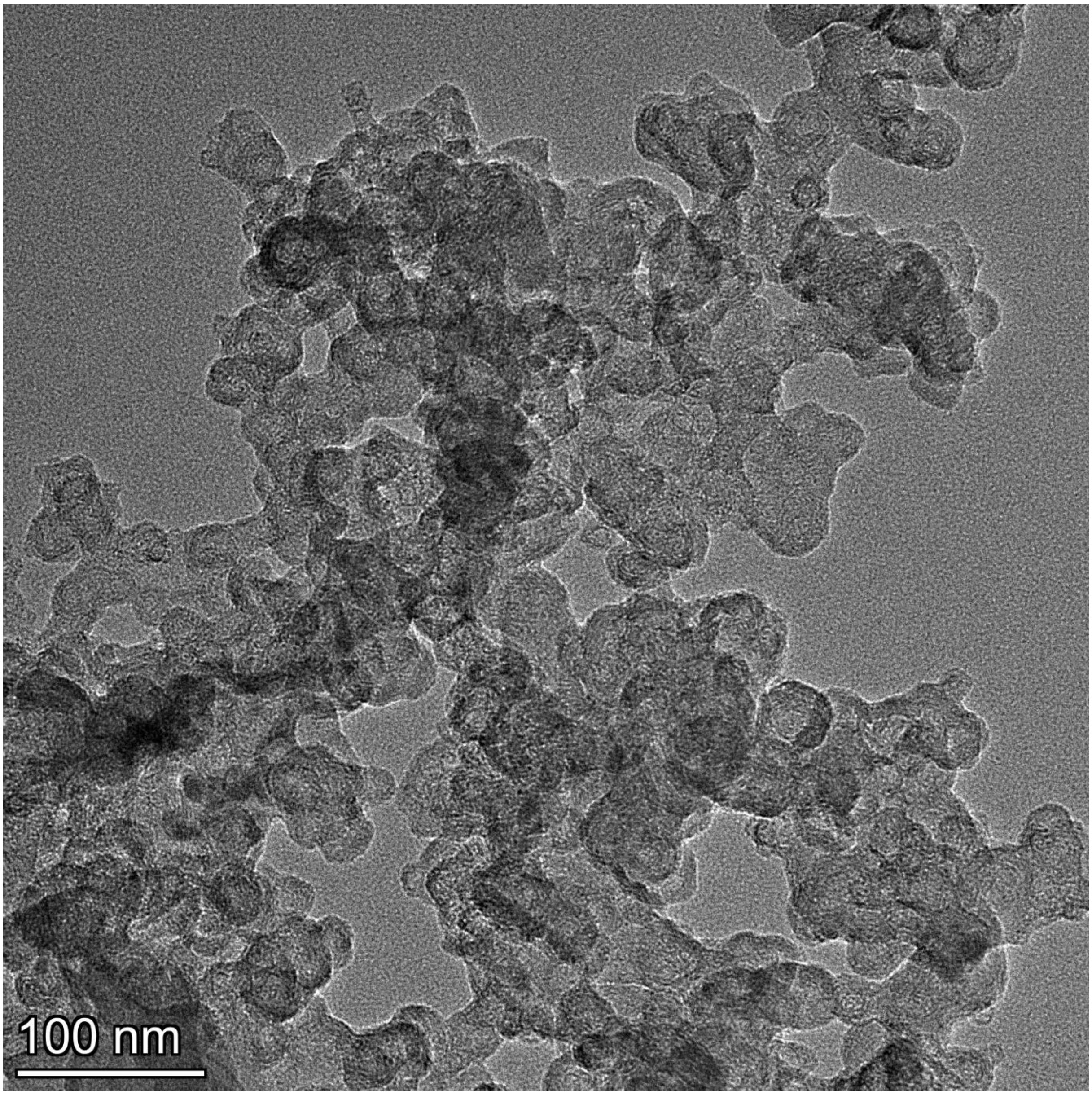

Figure S2. TEM images of POMs at pH 5.5.

Table S1. The important infrared peaks corresponding to key functional groups of POMs, $MnBr(CO)_5$, and PMC.

Wavelength number (cm^{-1})	Assignment
3226	OH...H
1900-2100	-C=O
1670	H_2O
1476	NH_4^+
973, 886	Mo=O
561	O-Mo-O

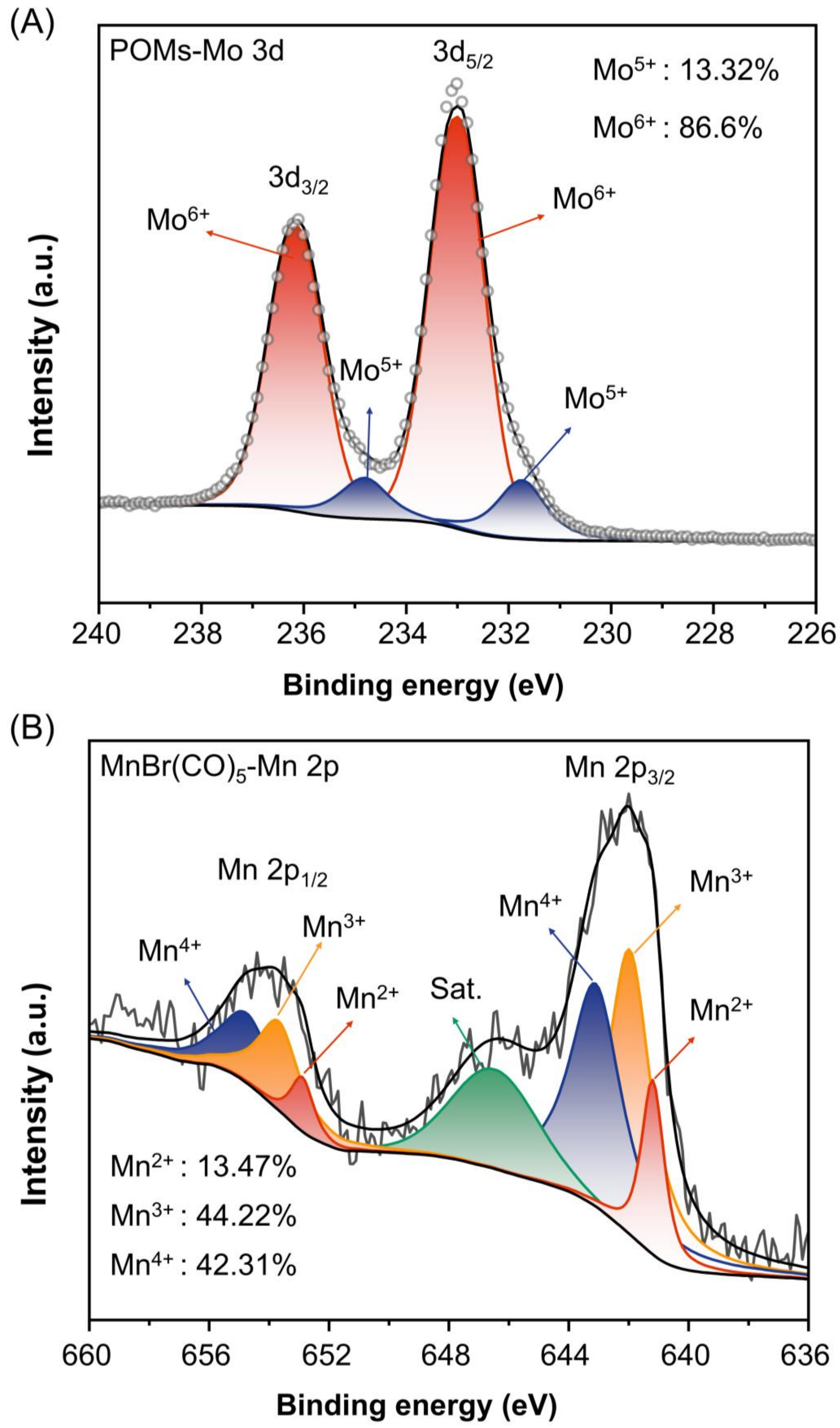

Figure S3. XPS spectra of Mo 3d for (A) POMs and Mn 2p for (B) $MnBr(CO)_5$.

Table S2. Quantification of Mo^{5+} and Mn^{2+} Proportions from XPS Analysis.

	Mo^{5+}	Mn^{2+}
POM	13.32%	/
$MnBr(CO)_5$	/	13.47%
PMC	17.06%	47.91%

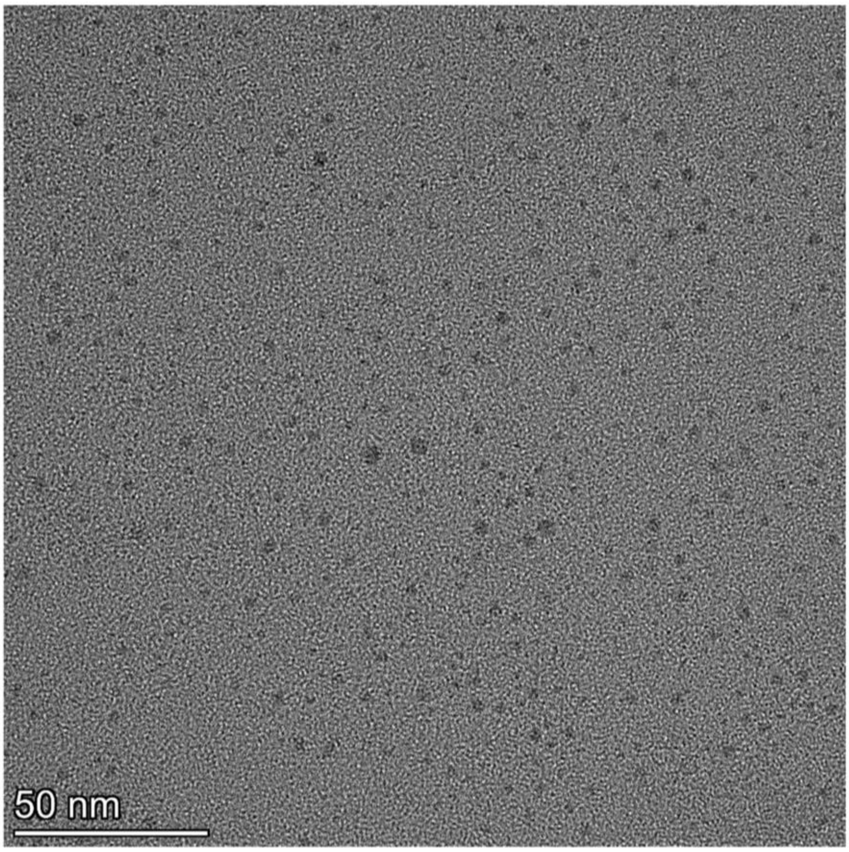

Figure S4. TEM images of POMs at pH 7.4.

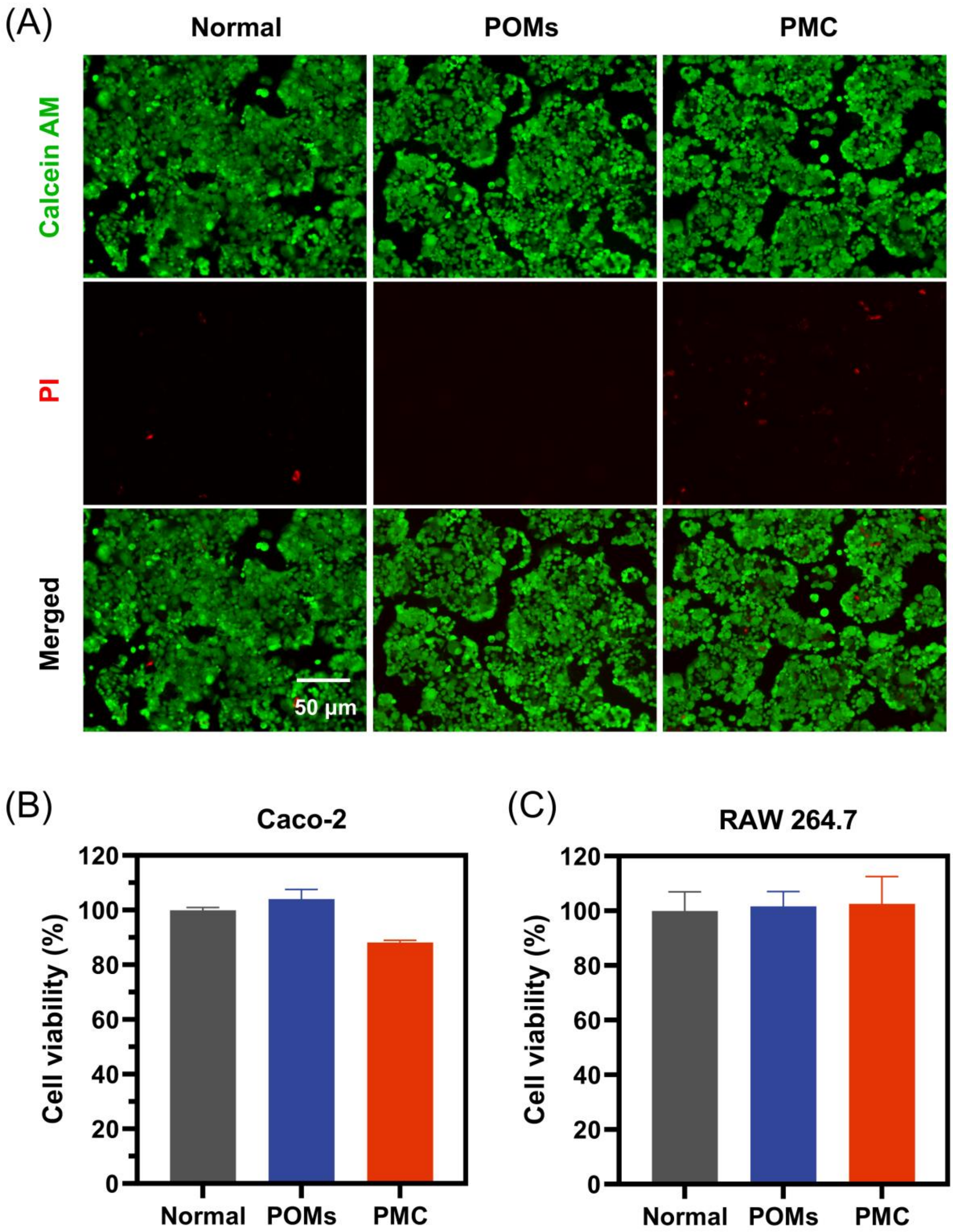

Figure S5. (A) Live/dead staining of Caco-2 cells. (B,C) Cell viability assay results for Caco-2 and RAW 264.7 cells.

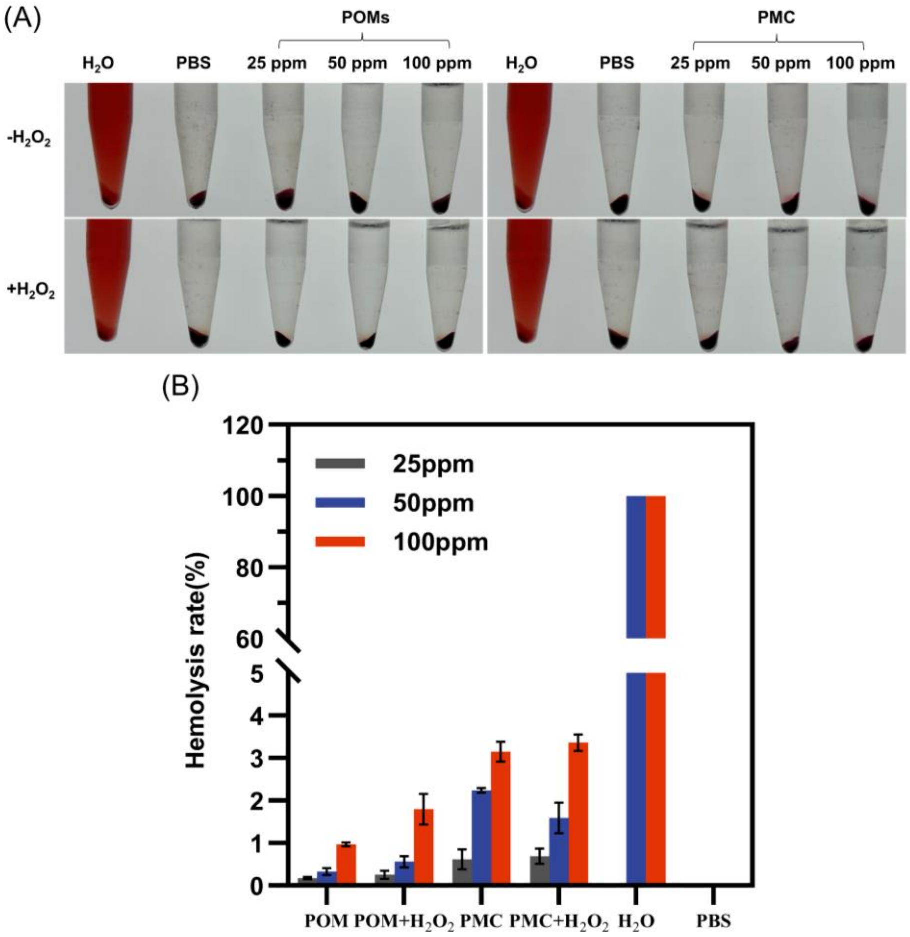

Figure S6. A) Hemolysis and B) hemolysis rates of POMs and PMCs.

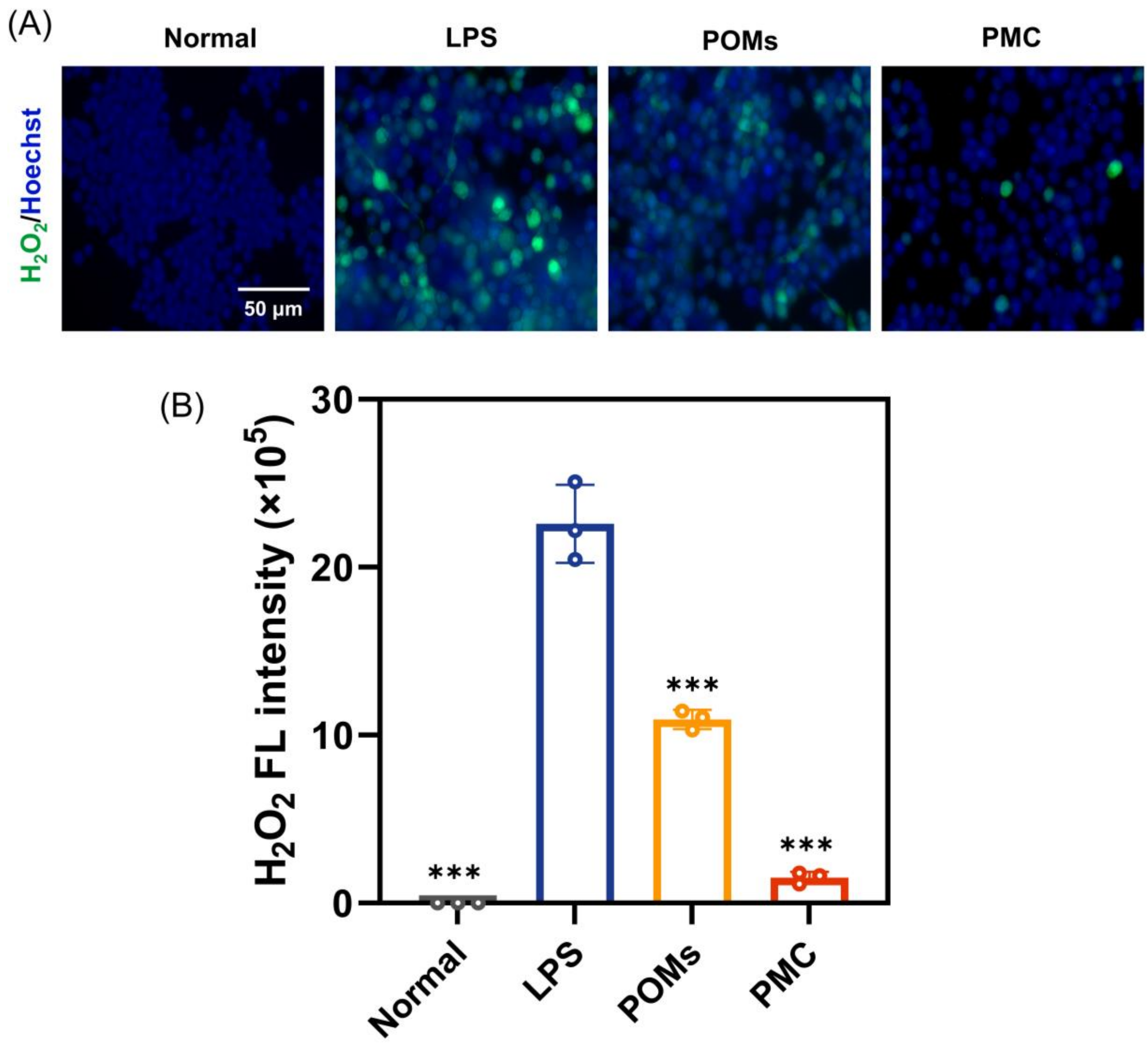

Figure S7. (A) Intracellular H_2O_2 imaging and (B) quantification of H_2O_2 fluorescence intensity in activated macrophages. Data are expressed as mean ± SD, n = 5, *** $p < 0.001$. Statistical comparisons were made between the LPS group and all other treatment groups.

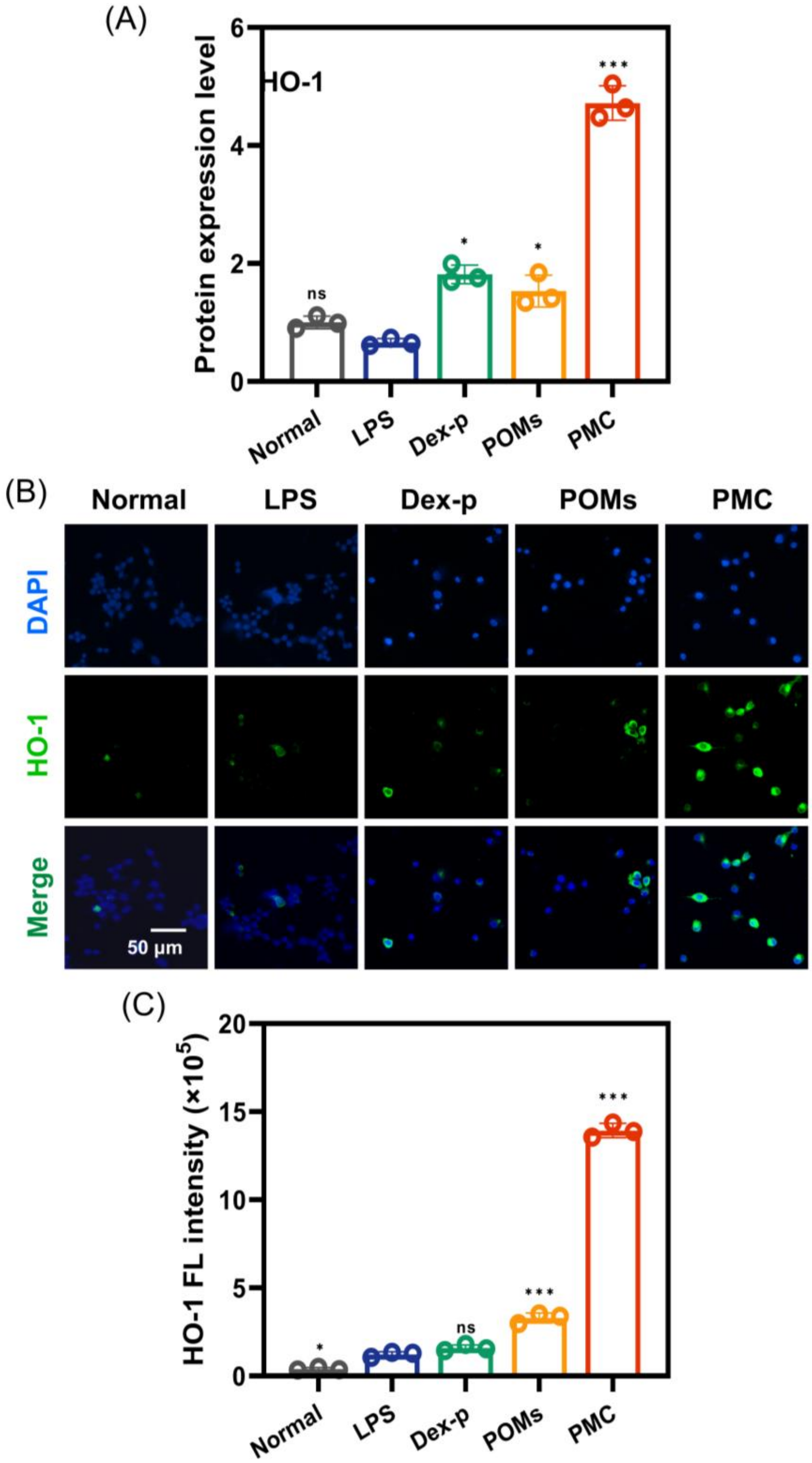

Figure S8. A) Protein expression levels of HO-1; B,C) Immunofluorescence staining of HO-1; Data are expressed as mean ± SD, n = 5, * $p < 0.05$, *** $p < 0.001$, ns: no significance. Statistical comparisons were made between the LPS group and all other treatment groups.

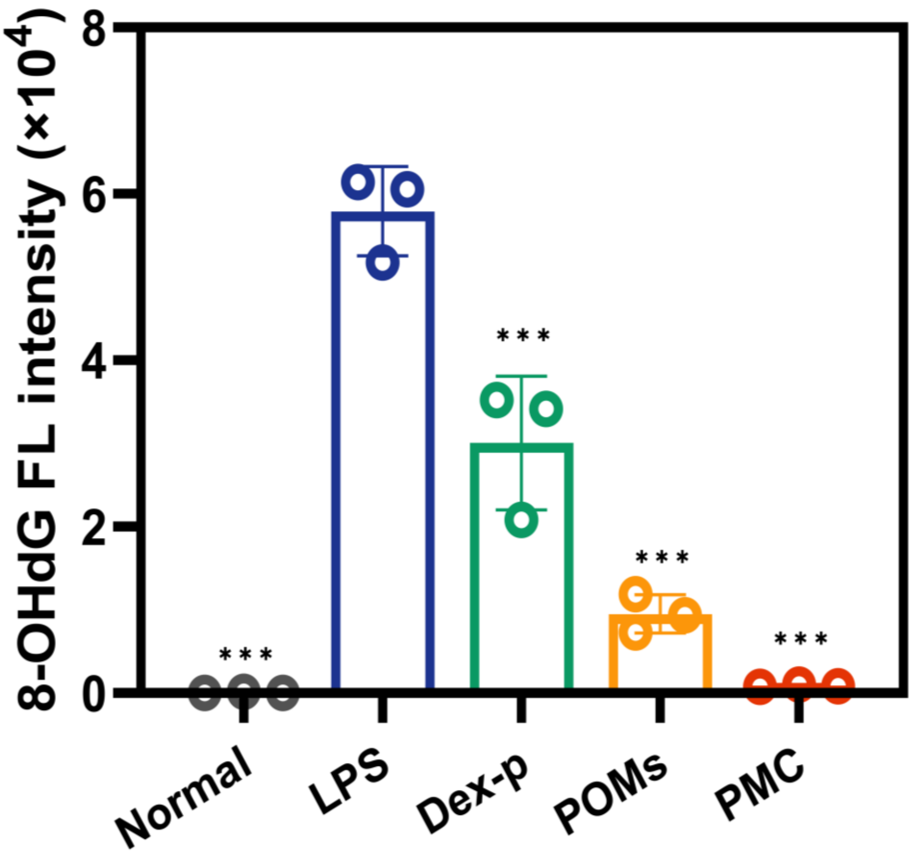

Figure S9. Quantification of intracellular 8-OHdG fluorescence after various treatments. Data are expressed as mean ± SD, n = 5, *** $p < 0.001$. Statistical comparisons were made between the LPS group and all other treatment groups.

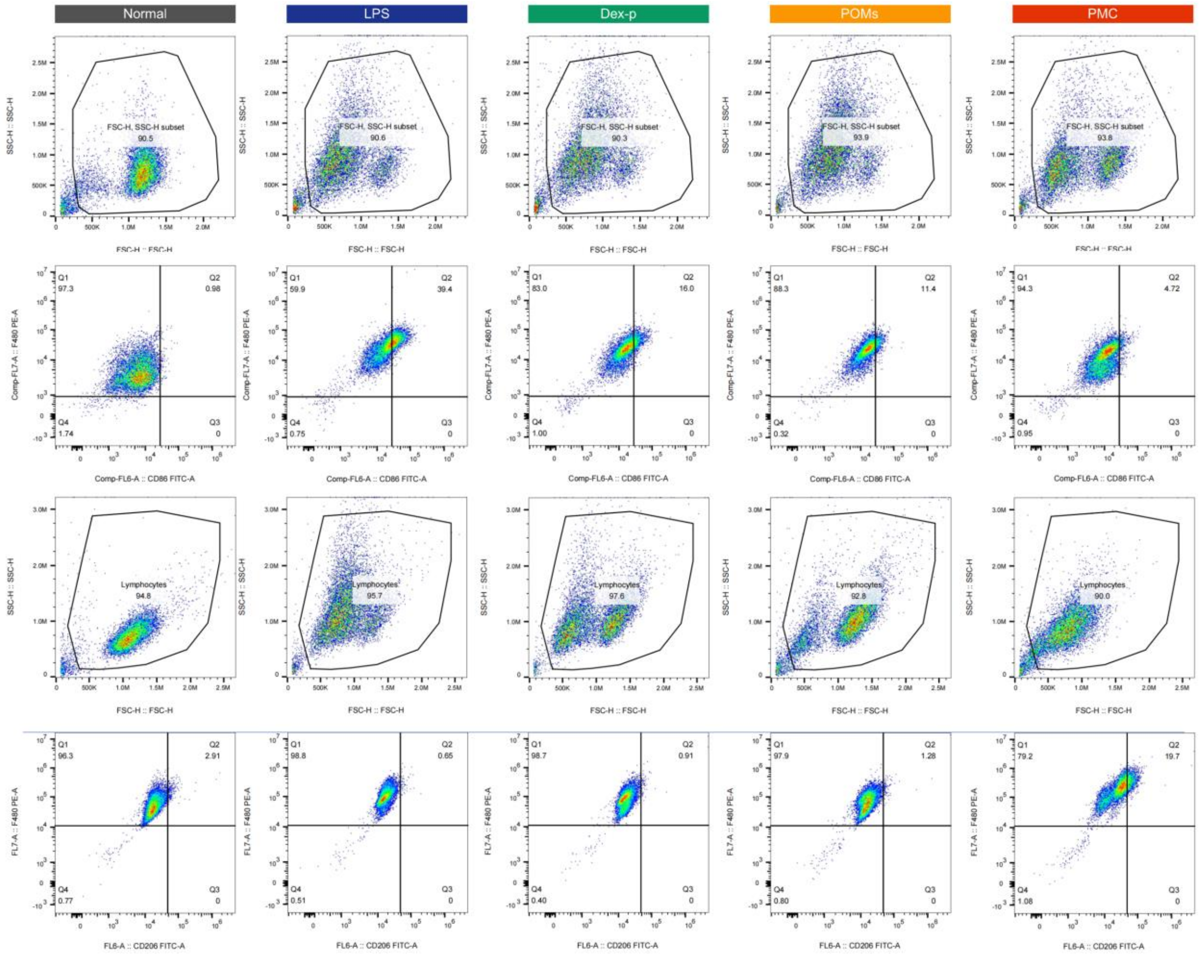

Figure S10. Flow cytometry analysis of macrophages phenotype following various treatments.

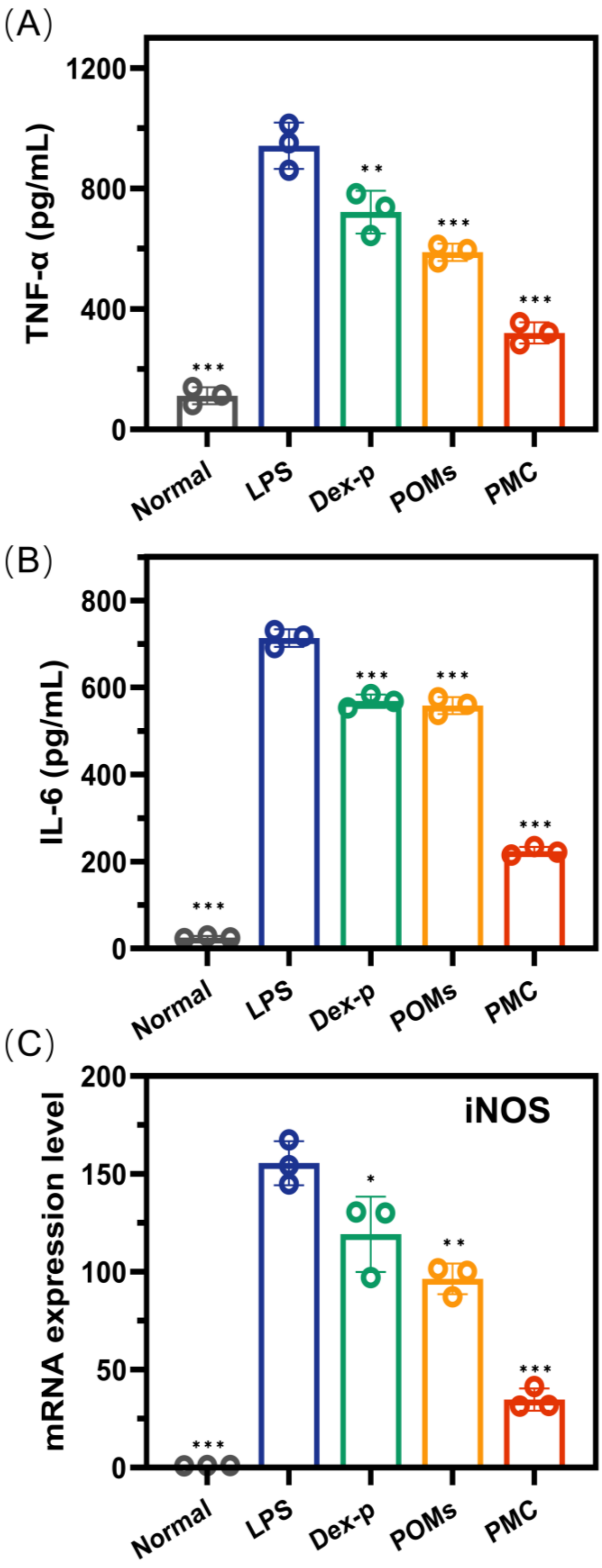

Figure S11. (A) mRNA expression levels of iNOS and protein expression levels of (B) TNF-α and (C) IL-6 in activated macrophages treated with PMC. Data are expressed as mean ± SD, n = 5, * $p < 0.05$, ** $p < 0.01$, *** $p < 0.001$. Statistical comparisons were made between the LPS group and all other treatment groups.

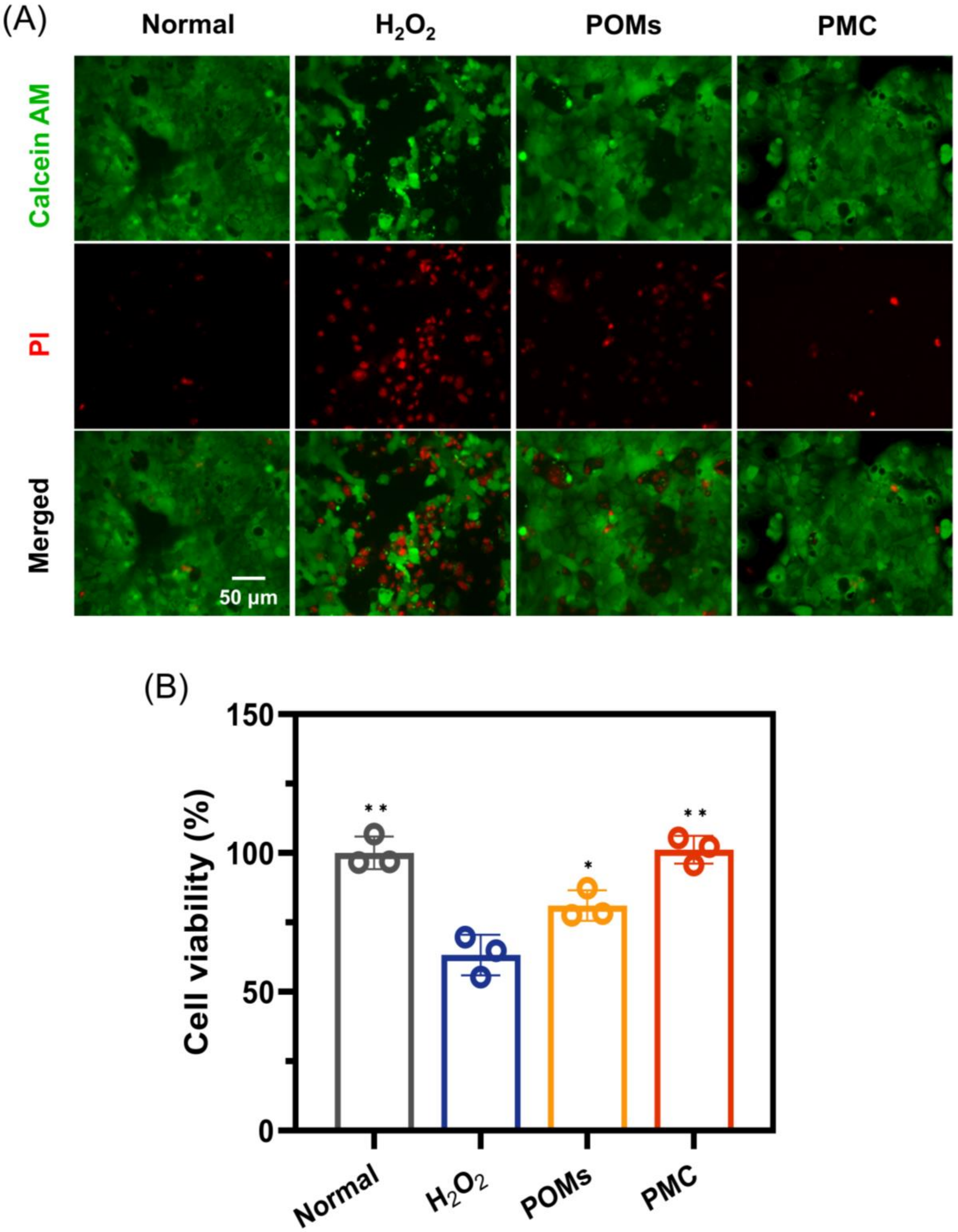

Figure S12. Live/dead staining and cell viability of Caco-2 cells upon exposure to 1 mM H_2O_2 for 20 h. Data are expressed as mean ± SD, n = 5, * $p < 0.05$, ** $p < 0.01$, *** $p < 0.001$. Statistical comparisons were made between the H_2O_2 group and all other treatment groups.

Table S3. The Primer sequence of genes used in the study.

Gene	Forward primer sequence (5'-3')	Reverse primer sequence (3'-5')
HO-1	AAGACTGCGTTCCTGCTCAAC	AAAGCCCTACAGCAACTGTCG
TNF-α	TCCAGGCGGTGCCTATGTC	CCTCCACTTGGTGGTTTGTGA
IL-1β	TGGAGAGTGTGGATCCCAAG	GGTGCTGATGTACCAGTTGG
IL-6	GCTGGAGTCACAGAAGGAGTGG	GGCATAACGCACTAGGTTTGC
iNOS	CGCTTGGGTCTTGTTCACT	TCTTCAGGTCACTTGGTA
CD206	GGACGAAAGGCGGGATGT	GGGTTCAGGAGTTGTTGTGGG
Arg-1	GTGAACACGGCAGTGGCTTTA	AGGCGTTTGCTTAGTTCTGTCTG
ZO-1	TCACGCAGTTACGAGCAAGT	TGAAGGTATCAGCGGAGGGA
Occludin	TCAGGGAATATCCACCTATCACTTCAG	CATCAGCAGCAGCCATGTACTCTTCAC
Claudin-1	GAAGTGCTTGGAAGACGATG	GAGCCTGACCAAATTCGTAC

Table S4. Content of Mo and Mn atoms in colonic tissues (μg/500 mg tissue) at 6- and 12-hours post-administration.

	Healthy+PMC		DSS+PMC	
	Mo	Mn	Mo	Mn
6 h	1.79	1.2	14.6	1.97
12 h	0.55	0.19	3.93	0.76

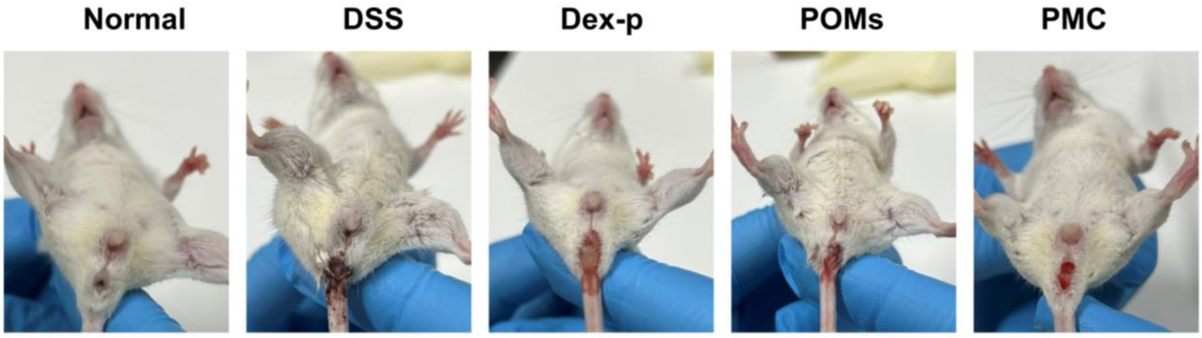

Figure S13. Occurrence of occult blood in colitis mice under various treatments.

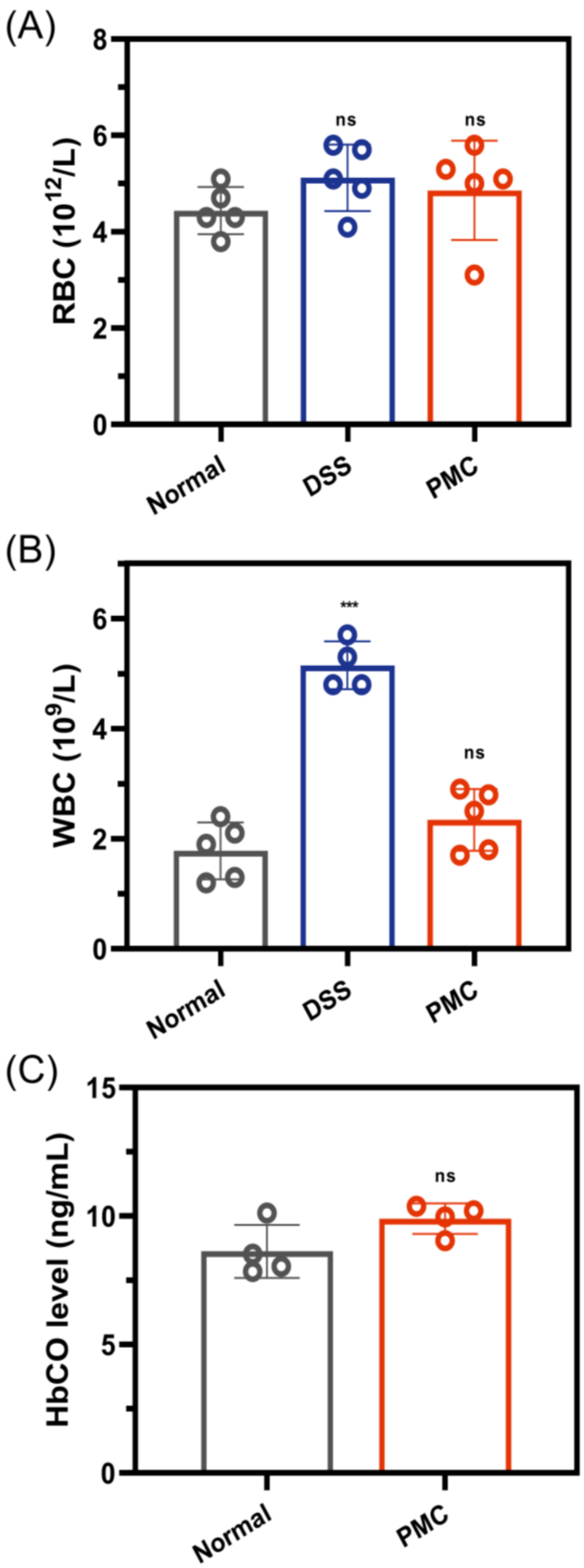

Figure S14. Blood cell counts, including RBC, WBC, and HbCO levels in colitis mice after various treatments on day 13. Data are expressed as mean ± SD, n = 5, *** $p < 0.001$, ns: no significance. Statistical comparisons were made between the normal group and all other treatment groups.

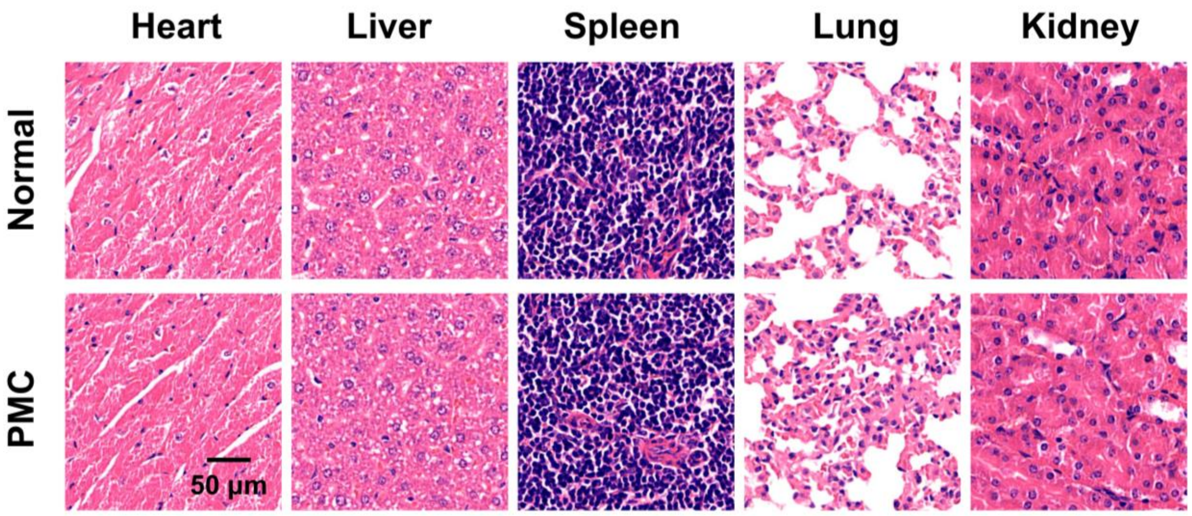

Figure S15 H&E staining of major mouse organs from the Normal and PMC groups on day 13.

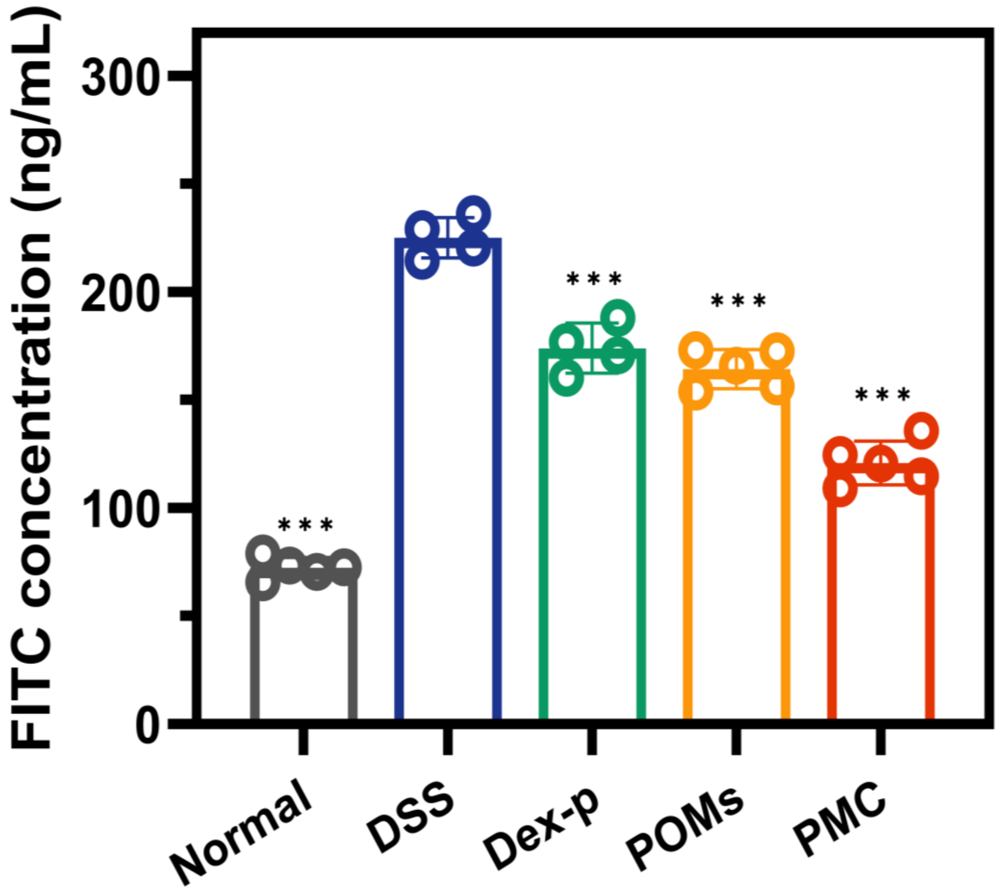

Figure S16 Concentration of FITC-dextran in the supernatant of blood collected from colitis mice. Data are expressed as mean ± SD, n = 5, *** $p < 0.001$. Statistical comparisons were made between the DSS group and all other treatment groups.

Table S5. Bacterial Classification of Pathogenic and Beneficial Bacteria at the Family and Phylum Levels.

Family		Phylum	
Pathogenic bacteria	Probiotics	Pathogenic bacteria	Probiotics
Enterobacteriacea	S24-7	Proteobacteria	Bacteroidetes
Enterococcaceae	Bacteroidaceae	Tenericutes	Firmicutes
Erysipelotrichaceae	Verrucomicrobiaceae	Cyanobacteria	Verrucomicrobia
Rikenellaceae	Lachnospiraceae	TM7	Actinobacteria
/	Lactobacillaceae	Fusobacteria	Deferribacteres
/	Ruminococcaceae	/	/

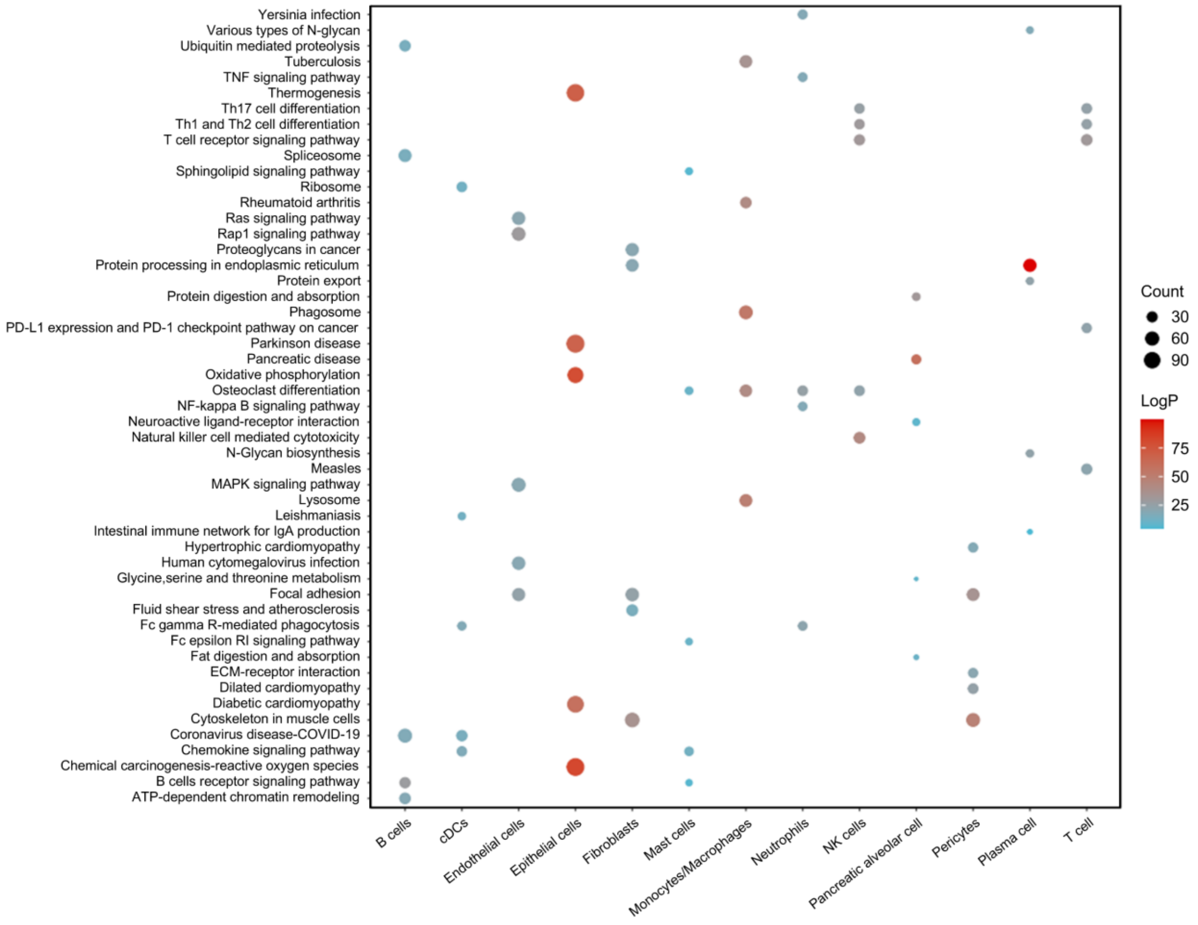

Figure S17 Analysis of the top five KEGG enrichments across different cellular clusters.

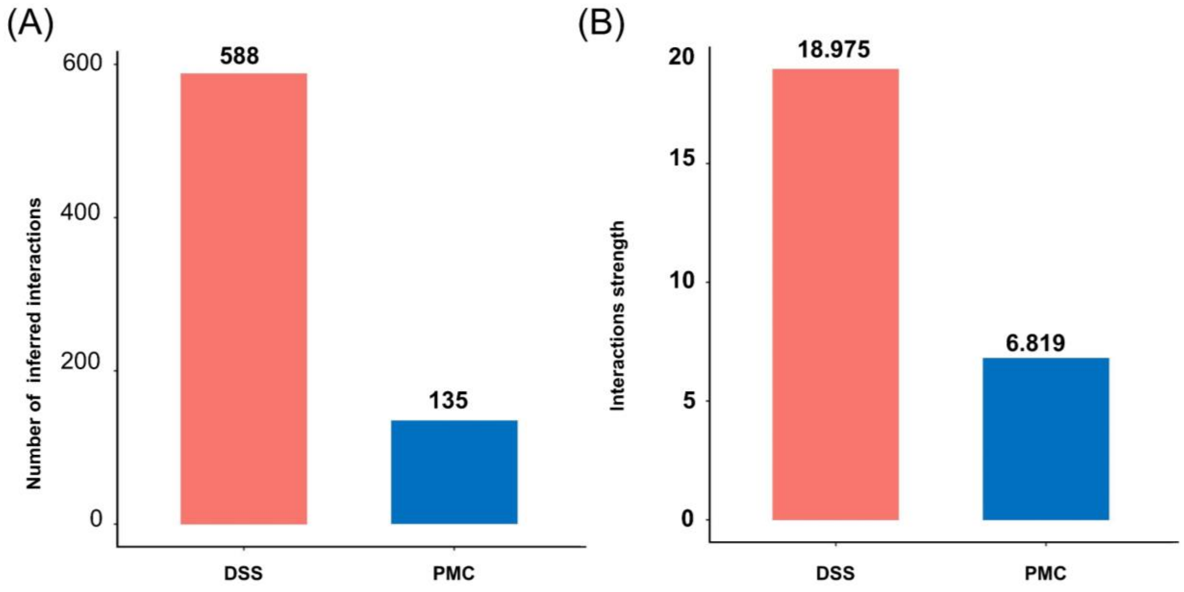

Figure S18 (A) The number of inferred interactions and (B) the interactions strength of the ligand–receptor in re-clustered Mon/Mac cells between PMC-treated group and DSS-treated group.

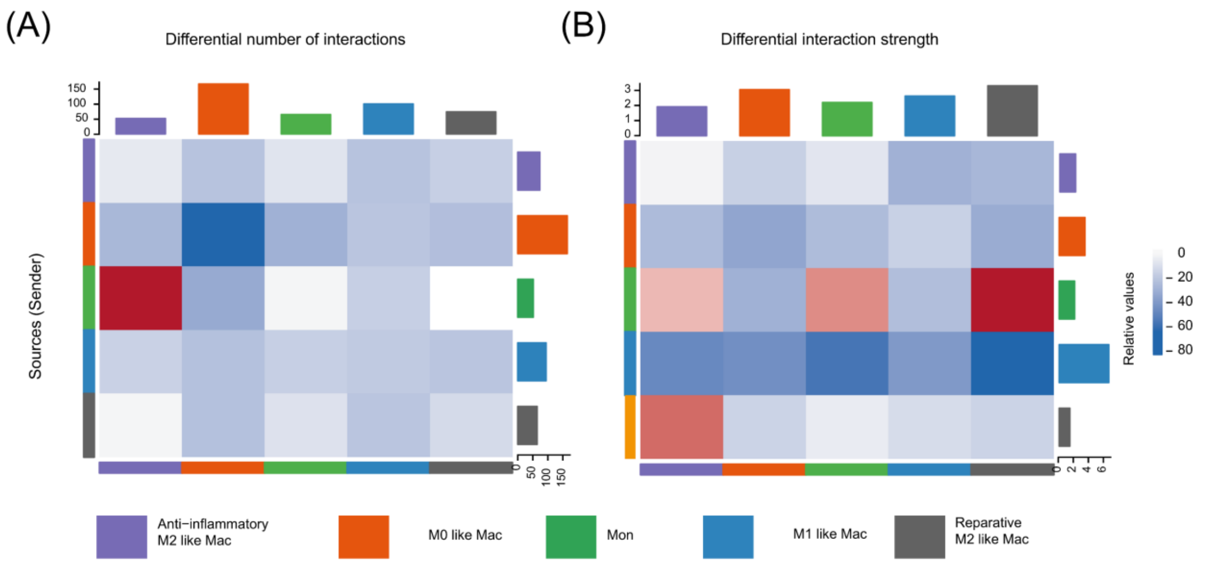

Figure S19 Heatmap showing the ligand–receptor pairs of differential interactions number and differential interactions strength in PMC-treated group compared DSS-treated group.

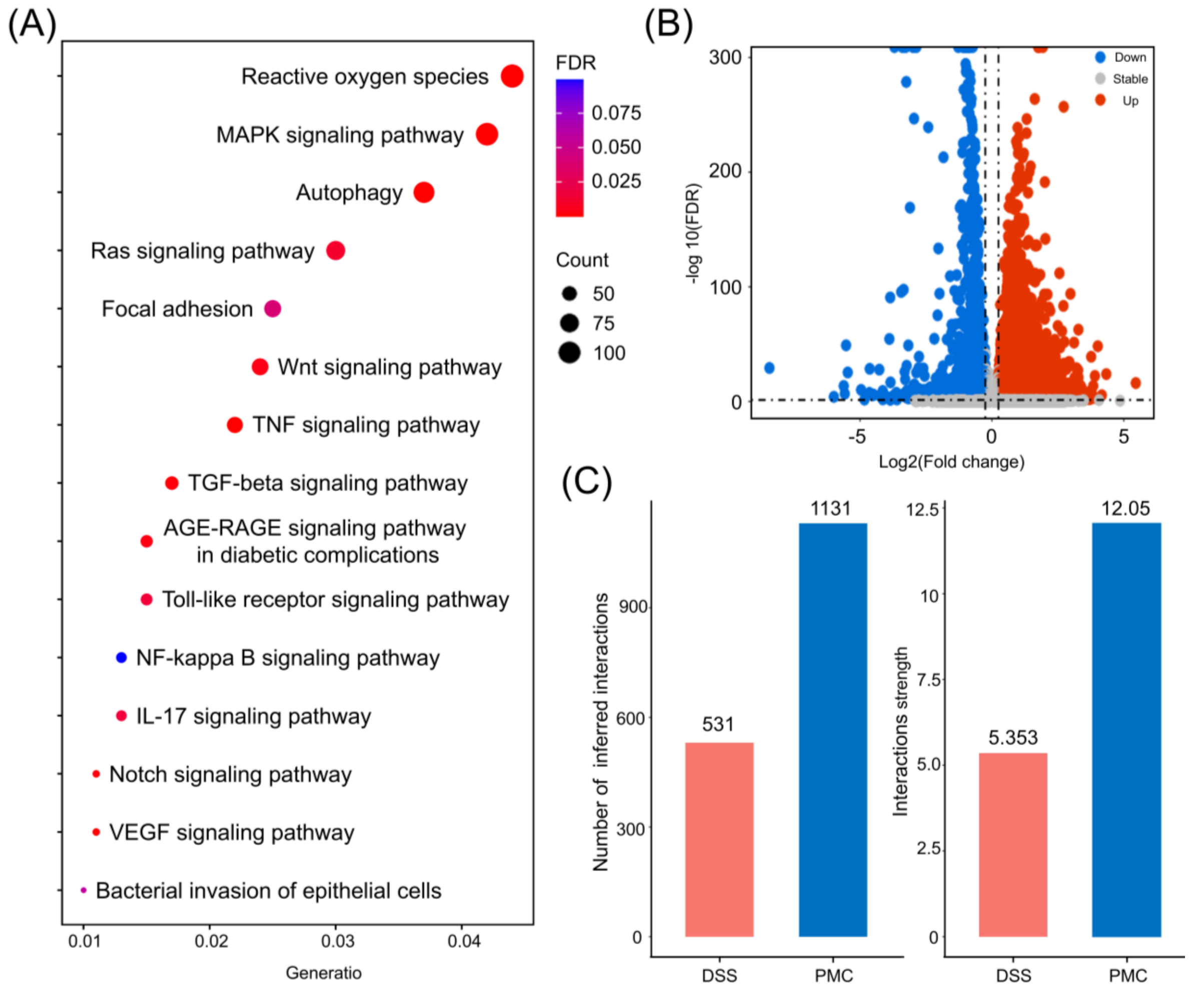

Figure S20 (A) Volcano diagram displaying DEGs in Epi cells between PMC-treated and DSS-treated group**.** (B) Kyoto Encyclopedia of Genes and Genomes (KEGG) analysis of Epi cells in PMC-treated group compared DSS-treated group. (C) The number of inferred interactions.

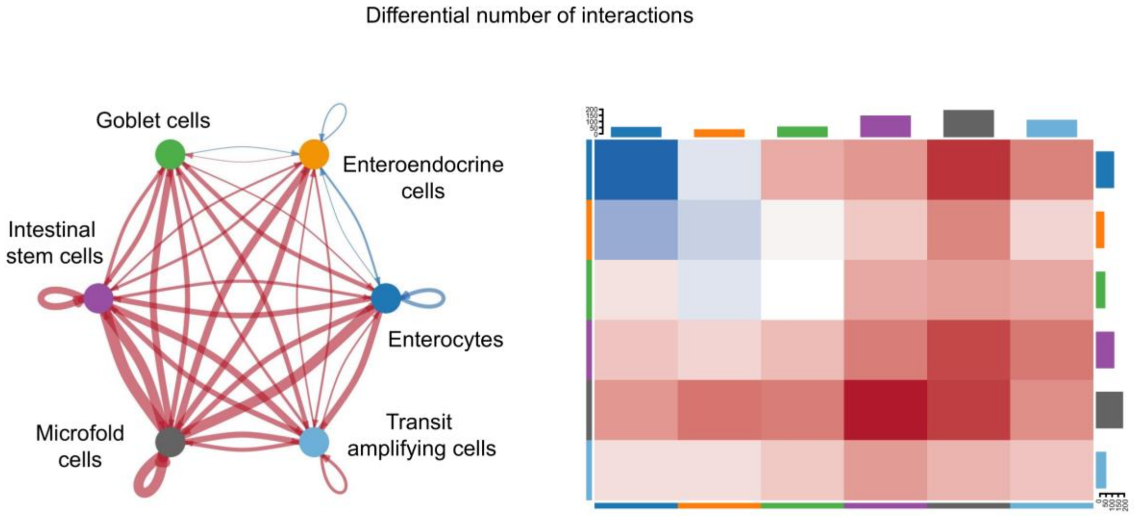

Figure S21 The number of inferred ligand–receptor interactions between the PMC-treated and DSS-treated groups.
